# Eco-Metabolomics Applied to the Chemical Ecology of Poison Frogs (Dendrobatoidea)

**DOI:** 10.1007/s10886-023-01443-0

**Published:** 2023-08-18

**Authors:** Mabel Gonzalez, Chiara Carazzone

**Affiliations:** 1 Department of Chemistry, Universidad de los Andes, 4976 Bogotá, AA Colombia; 2 Department of Biology, Stanford University, Palo Alto, CA 94305 USA

**Keywords:** Dendrobatoid, Amphibian, Chemical defences, Metabolomics, Chromatography, Mass spectrometry, Alkaloids

## Abstract

Amphibians are one of the most remarkable sources of unique natural products. Biogenic amines, peptides, bufodienolides, alkaloids, and volatile organic compounds have been characterized in different species. The superfamily Dendrobatoidea represents one of the most enigmatic cases of study in chemical ecology because their skin secretome is composed by a complex mixture (i.e. cocktail) of highly lethal and noxious unique alkaloid structures. While chemical defences from dendrobatoids (families Dendrobatidae and Aromobatidae) have been investigated employing ecological, behavioral, phylogenetic and evolutionary perspectives, studies about the analytical techniques needed to perform the chemical characterization have been neglected for many years. Therefore, our aim is to summarize the current methods applied for the characterization of chemical profiles in dendrobatoids and to illustrate innovative Eco-metabolomics strategies that could be translated to this study model. This approach could be extended to natural products other than alkaloids and implemented for the chemical analysis of different species of dendrobatoids employing both low- and high-resolution mass spectrometers. Here, we overview important biological features to be considered, procedures that could be applied to perform the chemical characterization, steps and tools to perform an Eco-metabolomic analysis, and a final discussion about future perspectives.

**Graphical Abstract:**

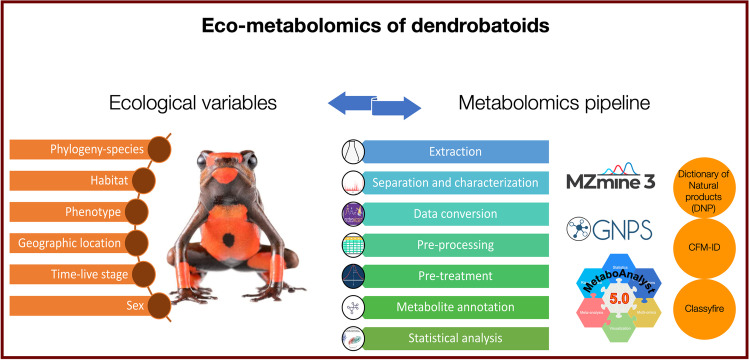

**Supplementary Information:**

The online version contains supplementary material available at 10.1007/s10886-023-01443-0.

## Introduction

### Purpose of this Review

We have reviewed the literature published from 1963 to 2023 that described the analytical chemistry procedures to characterize and identify metabolites from dendrobatoids. We found that most of the chemical procedures were targeted for alkaloids and the most recent review on the chemical ecology of poison frogs was published in 2012. This work focused mainly on the ecology and biochemistry of toxicity. From 2012 to present there has not been significant updates in the analytical chemistry of poison frogs. Our goal is to review the chemistry aspect of the chemical ecology of poison frogs. This review will be a tool to bring the Metabolomics era to biologists and chemists interested in studying the unique chemodiversity found in the superfamily Dendrobatoidea and their ecology.

The superfamily Dendrobatoidea sensu Grant et al. 2017 is composed by two families: Dendrobatidae and Aromobatidae (Grant et al. 2017; Guillory et al. 2019).Most of the analyses performed on this superfamily were led by John Daly and colleagues, who set the bases for the isolation and characterization of the unique alkaloids found in frogs from this phylogenetic group (Daly 2003). Most of their methodologies have been maintained and only slightly modified for the past 47 years to perform the chemical characterization of different morphotypes and species. These studies allowed to discover that each frog contained a complex mixture of alkaloids, also known as alkaloid cocktail and led to the full elucidation of many structures. These discoveries led to significant advancements studying pharmacological properties and its potential therapeutical applications. However, the information about chemical characterization of metabolites from dendrobatoids is spread in publications made over more than 40 years and the last database for mass spectrometry data was published 16 years ago (Daly et al. 2005).

In recent years, the improvement of High-Resolution Mass Spectrometry (HR-MS) capabilities led to obtaining new chemical information through new ionization techniques, such as Electrospray Ionization (ESI) and Desorption Electrospray Ionization (DESI). At the same time, Metabolomics workflows set a basis for improving the quality of the chemical analysis performed decreasing the likelihood of pseudo-replication. Based on this workflow, emerged Eco-metabolomics. This is a trans-disciplinary research area that links biochemistry and ecology of organisms for analyzing large quantities of complex experimental data derived from chemical analysis. Studying interactions between organisms, or organisms with the environment in different spatiotemporal scales can be tremendously enhanced using this approach. The term is not yet established in the scientific community but differs from the traditional Metabolomics in the type of questions aimed to be answer. The goal of Eco-metabolomics is to understand ecological processes tracing ﻿biochemical evidence, usually using several non-model species where true biological replicates are more difficult to obtain (Peters et al. 2018). Unfortunately, less than a dozen of investigations have applied HR-MS to study the chemistry of poison frogs (Fitch et al. 2010; McGugan et al. 2016; Caty et al. 2019; Fischer et al. 2019; Protti-Sánchez et al. 2019; Jeckel et al. 2020; Martin et al. 2020; Moskowitz et al. 2020) and only some aspects from an Eco-metabolomic approach have been exploited (Saporito et al. 2006, 2007, 2010; Mina et al. 2015; Caty et al. 2019; Moskowitz et al. 2020; O’Connell et al. 2021). It is important to clarify that this approach could be applied to data obtained from both, Low- and High- resolution mass spectrometers. The main goals of this review are (1) to provide an overview about the current knowledge about the chemical characterization of metabolites from dendrobatoids, and (2) to propose the development of different procedures, analytical tools, and data processing methods from an Eco-metabolomics approach to characterize metabolites from dendrobatoids. Additionally, the implementation of an Eco-metabolomics pipeline holds immense potential to further enhance our understanding of the evolutionary physiology and ecology of poison frogs, particularly when integrated with genomics and transcriptomics.

### General Introduction

In 1963, Drs. Märki and Witkop, published the first work about the venom of the Colombian arrow poison frog, *Phyllobates bicolor* (Märki and Witkop 1963). Five years later, Tokuyama et al. (1968) published the first proposed structure of batrachotoxin, a steroidal lethal alkaloid (Tokuyama et al. 1968), breakthrough that allowed the discovery of more than 800 alkaloids found in the class Amphibia (Daly et al. 2005). During the first years, most of the studies were focused on the chemical characterization and elucidation of structures (Daly et al. 1965; Daly and Witkop 1971; Myers et al. 1978). Then, after acquiring a better knowledge about the structures found in this group of amphibians, pharmacological properties started to be researched (Kayaalp et al. 1970; Albuquerque et al. 1971; Honerjäger and Reiter 1977). The subsequent chemical analyses were (and still are mainly) oriented to answering evolutionary questions or to study their bioactivity (Daly et al. 1994c, b; Daly 1995), and less focused on optimizing their procedures in analytical chemistry. Since 1963, advances in liquid chromatography coupled to mass spectrometry (LC–MS) and gas chromatography coupled to mass spectrometry (GC–MS) instrumentation, their workflows, and strategies for data analysis have been exploited for the chemical characterization and structure elucidation of new molecules in model organisms. However, for non-model species, such as poison frogs, the higher sensitivity and capabilities of modern instruments has not been fully exploited. High-Resolution Mass Spectrometers and different strategies from Metabolomics workflows have been employed in just a few studies. The most distinctive features about an Eco-metabolomics approach involves: (1) usually an untargeted analytical methodology, (2) the aim to measure the highest amounts of metabolites possible, (3) the focus on extrinsic processes acting upon organisms at population and community scales (e.g., predation, climate change, competition), (4) the inclusion of an experimental design for manipulating (preferable) or measuring (when is not possible to manipulate) variables different from metabolites (metavariables), (5) the traditional steps followed in any Metabolomic analysis (Peters et al. 2018), (e.g., extraction, data conversion, pre-treatmement, and metabolite annotation. For more details see Sect. 3). In order to have a better understating about the chemical ecology of poison frogs, all these steps could and should be applied for studying the chemical profiles of poison frogs obtained by Low- and High- Resolution LC–MS and GC–MS instruments.

### Chemodiversity in the Dendrobatoidea Superfamily

Amphibians exhibit a high diversity of natural compounds. Biogenic amines, peptides, proteins, bufadienolides and more than 900 alkaloids (Daly et al. 2005; Jones et al. 2012) have been found in the skin of different species. The Dendrobatoidea superfamily (Grant et al. 2017), exclusive to the Neotropics, is the group of amphibians with the highest abundance of alkaloids. More than 523 of them are lipophilic (Saporito et al. 2012), three are hydrophilic (Daly et al. 1994a) and some derived from biogenic amines have been putatively annotated (﻿only to MS^1^ level) recently on *Phyllobates vitattus* (Protti-Sánchez et al. 2019). The chemical analysis of non-alkaloid compounds, such as proteins (Caty et al. 2019; O’Connell et al. 2021; Alvarez-Buylla et al. 2022, 2023), peptides, volatile organic compounds (VOCs) (Gonzalez et al. 2021), lipids, pigments (Crothers et al. 2016; Twomey et al. 2020b, a), among others, has been almost neglected in this superfamily. This was likely influenced by the initial focus on studying chemical defences in dendrobatoids, where alkaloids were expected to be the primary contributors. However, the traditional perspective of chemical ecology, which aims to explore the variety of natural products in order to identify the specific compounds responsible for anti-depredatory defenses, is now being questioned by the Eco-metabolomics approach. This viewpoint has also faced challenges in the study of other organisms, as numerous compounds have been discovered to have multifunctional roles across different spatial and temporal scales, and they appear to be involved in multiple interactions among organisms (Raguso et al. 2015). Lipophilic molecules have been detected in many genera of the superfamily, while the hydrophilic tetrodotoxins (TTXs) have only been found in two species of the genus *Colostethus* (*C. panamensis* and *C. ucumari*) (Daly et al. 1994a; Grant 2004, 2007). The diversity of lipophilic alkaloids is so vast that a taxonomy of families of alkaloids has been created, including batrachotoxins (Daly et al. 1965; Tokuyama et al. 1968; Daly and Witkop 1971; Myers and Daly 1976a; Myers et al. 1978), histrionicotoxins (Daly et al. 1971; Myers and Daly 1976a), gephyrotoxins (Tokuyama et al. 1974; Daly et al. 1977), pumiliotoxins (Daly and Myers 1967; Myers and Daly 1976a, b; Daly et al. 1980; Tokuyama et al. 1984), allopumiliotoxins (Daly et al. 1980), homopumiliotoxins (Tokuyama et al. 1987), decahydroquinolines (Daly et al. 1969), pyrrolizidines (Garraffo et al. 1993), indolizidines (Daly et al. 1978; Spande et al. 1981), lehmizidines (Daly et al. 1986), pyrrolidines (Daly et al. 1986), piperidines (Daly et al. 1986; Edwards et al. 1988), quinolizidines (Jones and Gorman 1999), and pyridinic alkaloids as epibatidine (Spande et al. 1992) (Table 1). The most important discoveries and conditions employed for the structure elucidation of the alkaloid families found in dendrobatoids are summarized in Online Resource 1.

**Table 1:**
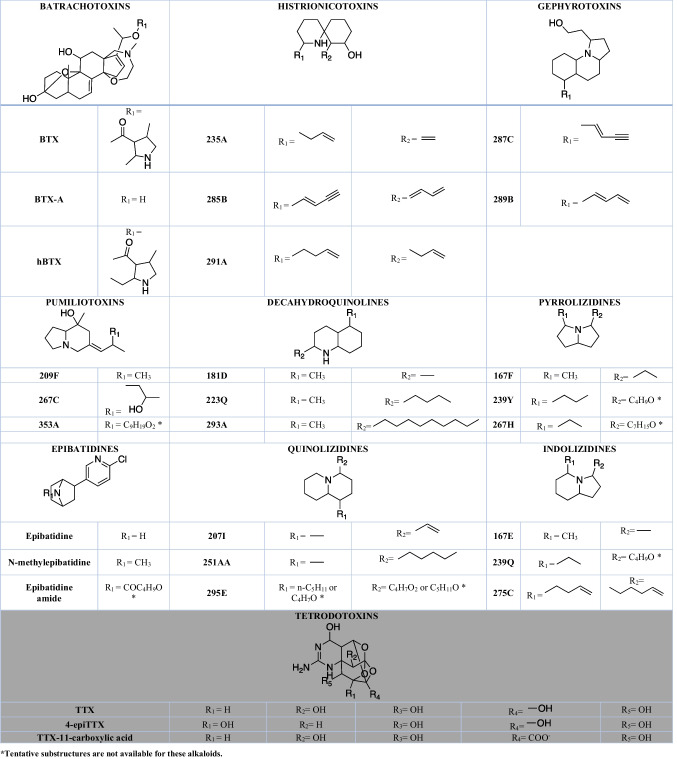
Families of lipophilic (white background) and hydrophilic (gray background) alkaloids from poison frogs from the Dendrobatoidea superfamily and three correspondent examples of alkaloids with low, medium, and high molecular weight. Structures obtained from Daly et al. (2005) and Asakawa et al. (2012) (Daly et al. 2005; Asakawa et al. 2012). Name of some alkaloids have a code name (bold-faced number + bold-faced letter) ranging from the lower nominal mass to the higher. The letter is organized alphabetically to differentiate individual alkaloids with the same nominal mass (For more details see Section "Data derived from GC–MS").

Dendrobatoids acquire these diverse alkaloids by three mechanisms: de novo biosynthesis, direct sequestration, or metabolic transformation of compounds ingested in the diet (Mebs 2001). In the case of alkaloids derived from biogenic amines in *Phyllobates vitattus* (Protti-Sánchez et al. 2019), they probably come from the biosynthesis of tryptophan as in bufonids (Scott Chilton et al. 1979). In contrast, for most of the lipophilic alkaloids, a dietary intake of precursors that may or may not undergo metabolic transformations has been proven (Daly et al. 2003, 2009; Santos et al. 2016; Jeckel et al. 2022). Inter-individual variation among species, populations, and specimens probably depends on genetic/transcriptomic differences that delimit resistance to dietary toxins (Santos et al. 2016; Tarvin et al. 2016), different metabolic capabilities (Abderemane-Ali et al. 2021; Márquez 2021), and the heterogeneous occurrence of dietary items on the forest in space and time (Saporito et al. 2012).

The origin of hydrophilic alkaloids in dendrobatoids remains unsolved. In other organisms that contain TTX it has been found that the origin of toxicity could be attributed to different bacteria from their microbiome (Hanifin 2010). Even in other amphibians, four different bacteria strains isolated from newt’s skin produce TTX *(﻿Aeromonas, Pseudomonas, Shewanella*, and *Sphingopyxis*) (Vaelli et al. 2020), but previous microbiome studies in dendrobatoids have failed detecting TTX (Martin et al. 2020).

### What is a “Poison Frog”?

**Fig. 1:**
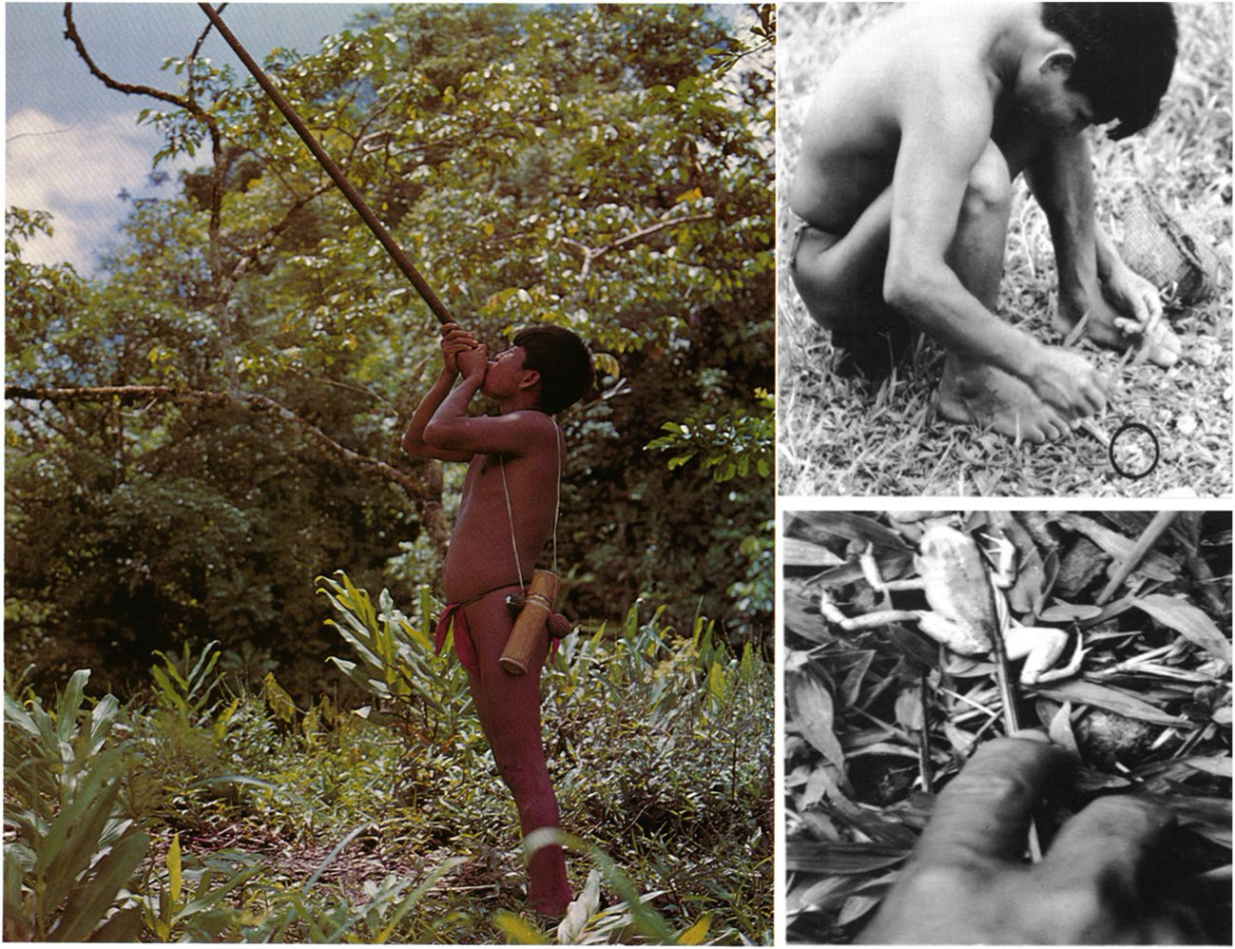
Emberara-siapidara Indians from Colombia using blowguns with darts poisoned with *P. terribilis* toxin. Photography was taken from the original article by Myers et al (1978). Copyright © 1978, Reproduction permit granted by the Natural History Museum of New York

Kokoi is the name of “a substance of unusually high toxicity which was discovered by the Indigenous people from Chocó in Colombia (South America)” (Märki and Witkop 1963). Emberá Indians from the department of Chocó and Risaralda discovered that they could poison darts with skin secretions from *Phyllobates bicolor* and use them to hunt their food (Märki and Witkop 1963). The name kokoi was used to name the toxin and the frogs. Later, Takashi Tokuyama, Charles Myers, John Daly, and Borys Malkin found that the Noamá, Cholos, and Emberá from the department of Chocó, and Emberara siapidara from Cauca, also poisoned their darts with *Phyllobates terribilis* and *P. aurotaenia* (Tokuyama et al. 1968; Myers et al. 1978). Actually, these studies found that *P. terribilis* is the most toxic vertebrate on earth. This etnopharmacological work and the way how indigenous people used the venom of these animals inspired to Märki and Witkop to call them “arrow poison frogs” or “poison dart frogs” (Myers et al. 1978). However, the authentic “poison dart frogs” are only three endemic frog species from Colombia used in the past by indigenous people for dart envenomation and traditional use with blowguns (for some illustrative examples see Fig. 1). Nowadays blowguns fabrications have been replaced by fire guns, and blowguns and darts are decorative or handcrafted items for sale, therefore frogs are not used for dart poisoning anymore. After more than 40 years of this collaborative work between biologists and chemists, more poisonous species were discovered and the terms “dart frog” or “poison dart frog” was erroneously extended to other species, and even used as a synonym of the complete Dendrobatoidea superfamily (known also as Dendrobatidae family before 2017). For the purpose of this review, we will utilize the term "poison frog" to encompass the entire superfamily Dendrobatoidea, but distinguishing as true “poison dart frogs” only the three Colombian *Phyllobates* species that were actually used for the indigenous communities for dart-envenomation.

### Phylogenetic Relations and Knowledge about Chemical Profiles

**Fig. 2:**
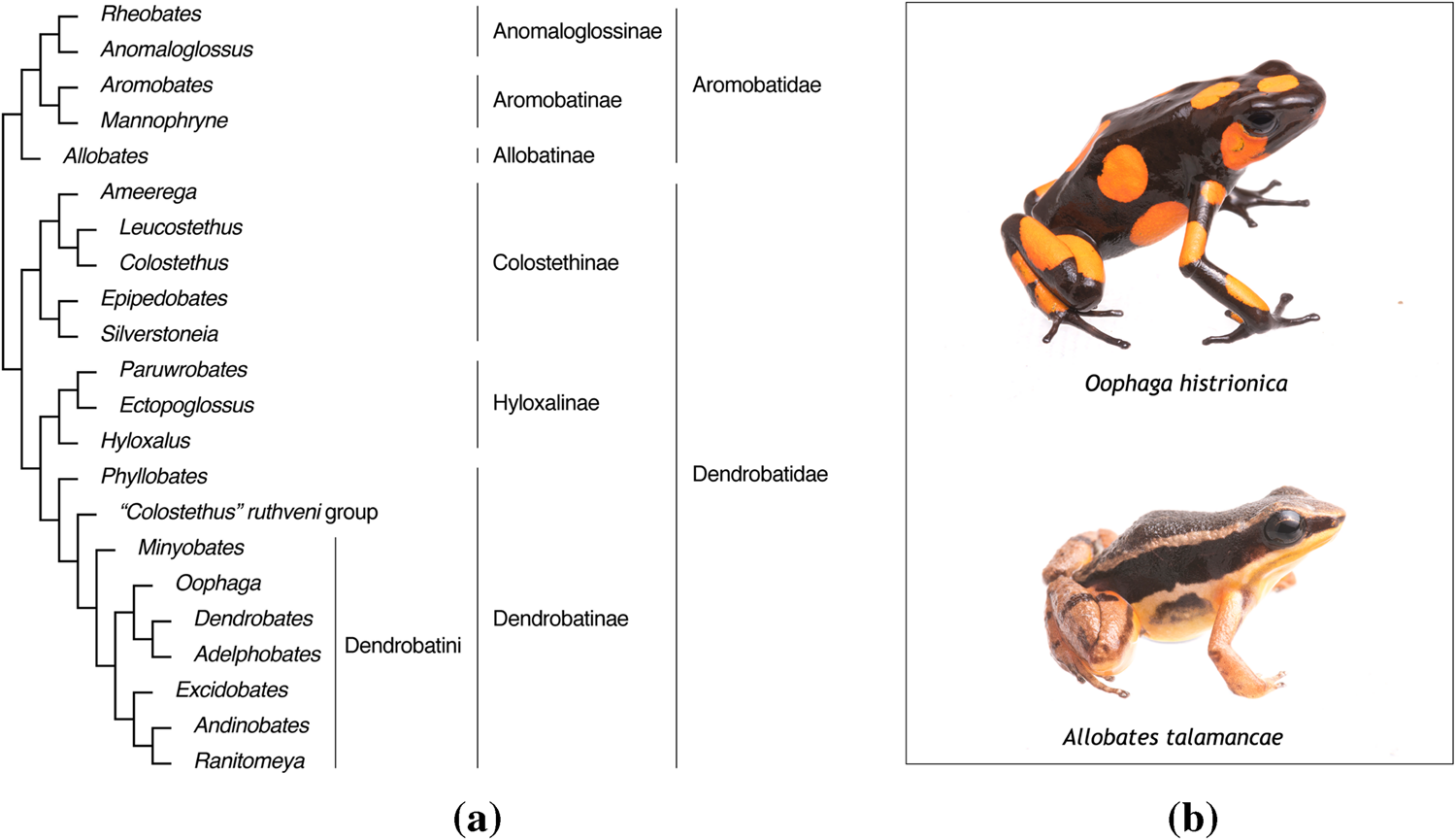
Generalities about Dendrobatoidea superfamily. **A**. Taxonomy of Dendrobatoidea superfamily. Copyright © 2017, kindly updated and shared by Grant et al. from (Grant et al. 2017). **B**. Photographies comparing a conspicuously colored dendrobatoid species (specimen: *Oophaga histrionica*) with a cryptic, brown coloration (specimen: *Allobates talamancae*). Photos by Sebastian Didoménico

Dendrobatoidea sensu Grant et al. 2017 superfamily consists of 22 genera (Grant et al. 2017; Guillory et al. 2019) divided in two families: Dendrobatoidae and Aromobatidae (see Fig. 2A). Seventeen of them belong to Dendrobatoidea and most of them are conspicuously colored. Five of them belong to Aromobatidae, which are largely considered cryptically colored (brownish) and to lack chemical defences (Grant et al. 2006) (see Fig. 2B for an illustrative example about differences in coloration). Cryptic species are found within both families and comprehend more than 200 species. The current knowledge states that within Dendrobatoidea, there are some chemically defended species and others where alkaloids are absent (Santos and Cannatella 2011; Santos et al. 2016). It has been generally assumed that the absence of alkaloids is equivalent to being chemically undefended. However, the discovery of metabolites other than alkaloids and the chemical characterization of unstudied taxa will demonstrate that we need to expand our definition about what chemical defences in dendrobatoids are. Also, there is a gap of knowledge of the chemical profiles of about 88% (176 species) of less colorful cryptic species (Santos et al. 2016). This causes that current evolutive correlations between toxicity and phenotypic traits of colored and cryptic dendrobatoid species to be biased. Only some *Aromobates*, *Colostethus*, *Epipedobates* and *Hyloxalus* are recognized to be chemically defended. Chemical analysis from most of the species of the genera *Rheobates*, *Anomaloglossus*, *Aromobates*, *Mannophryne*, *Allobates*, *Hyloxalus*, *Silverstoneia*, *Leucostethus*, and *Colostethus* have not yet been carried out (see Fig. 21.1 in Santos et al. 2016 for details about sampling coverage within each genus and alkaloids detected).

### Habitats and Microhabitats

The habitats where dendrobatoids live are found primarily associated with humid forests, at lowland distributions with altitudes of less than 2000 m. Almost all species are active during the day, unlike most amphibians that exhibit nocturnal peaks of activity (Santos et al. 2016). *Aromobates nocturnu*s, is one of the few exceptions that are active at night (Myers et al. 1991). Males actively defend their territory, while females often move between several male territories (Wells 2007).

### Aposematic Syndrome

**Fig. 3:**
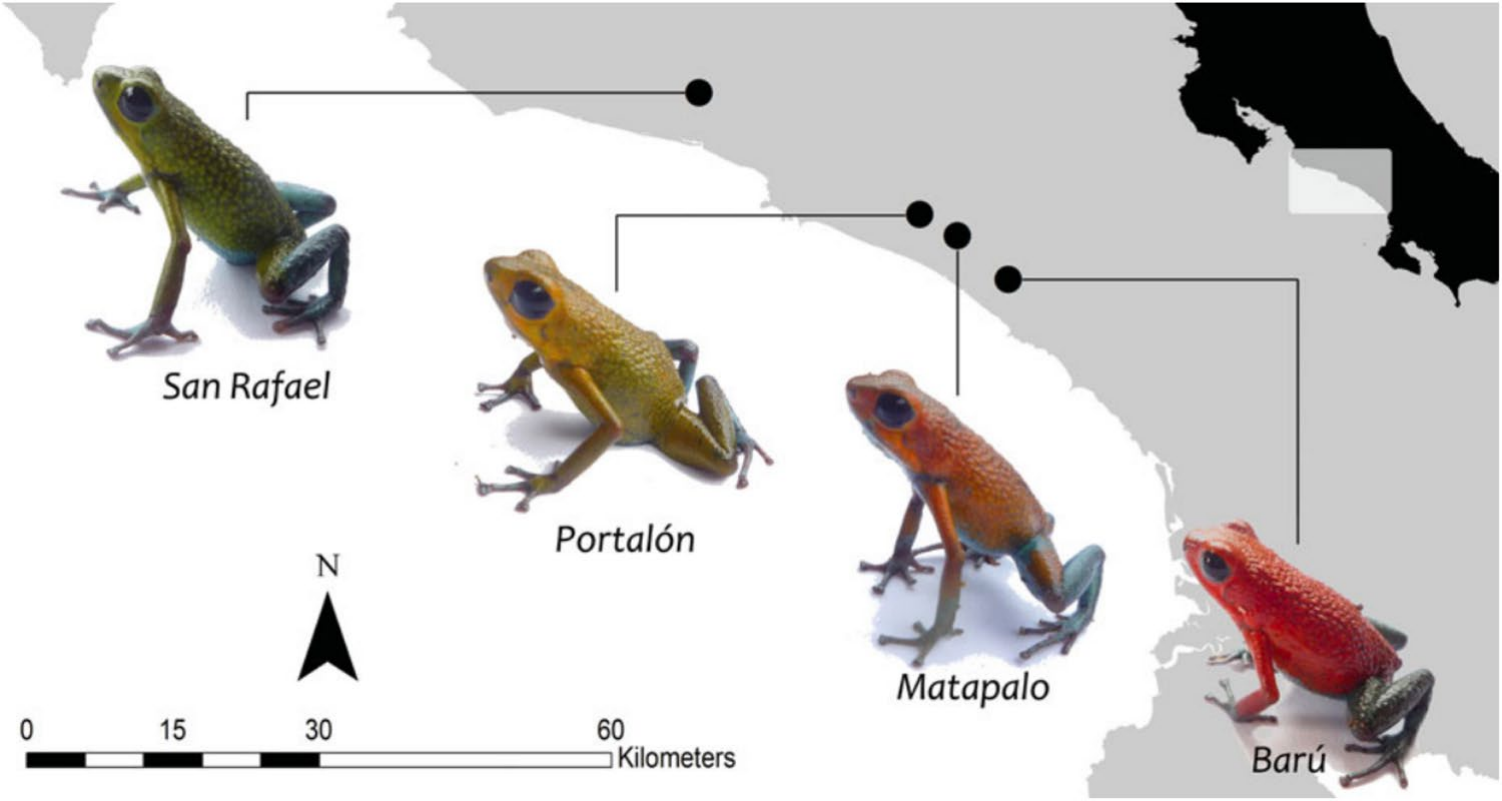
Polymorphic phenotypes of four populations of *Oophaga granulifera* and their geographic locations in the western lowlands of Costa Rica. Copyright © 2013, Reproduced from (Willink et al. 2013)

Aposematism is the co-ocurrence of warning signals and defence mechanisms (Sillen-Tullberg and Bryant 1983). This phenomenon has one of the most extravagant and interesting expressions on Dendrobatoidea superfamily and led to probably one of the major research fields in dendrobatoids (Santos and Cannatella 2011; Willink et al. 2013; Rojas et al. 2014, 2018; Santos et al. 2014; Galeano and Harms 2016; Carvajal-Castro et al. 2021). In general, it has been found that colorful coloration of some dendrobatoids could function as a warning visual signal of toxicity/unpalatability to predators (Santos et al. 2014). The relation between chemical defences and other phenotypic traits such as body mass, diet specialization, metabolic rate, and even coloration, have found that different “rules” applies to different species. An additional intriguing feature is that polymorphic phenotypes with different colorations within a single species are very common in this superfamily. An example of this variation is shown in Fig. 3 illustrating four *Oophaga granulifera* phenotypes from Costa Rica. This adds extra difficulties for studying aposematism, because warning coloration sometimes co-varies directly with chemical defences (Vences et al. 2003; Darst et al. 2005), other times co-varies inversely (Wang 2011), and other times there is no correlation (Darst et al. 2006).

Most of the initial findings within the superfamily involved toxic species that displayed conspicuous coloration (Summers and Clough 2001). Consequently, a majority of the research conducted thus far focused on examining ecological theories related to aposematism in colorful species. However, it should be noted that around two-thirds of the species in the Dendrobatoidea group exhibit cryptic coloration (Santos et al. 2016), and it is within this particular group where our understanding of their chemical profiles remains limited.

### Inter-Individual Variation in Alkaloid Profiles

Variation in alkaloid profile composition (number of alkaloids, type of alkaloids, quantity of alkaloids) has been documented since Daly et al. earliest work (Daly et al. 1987). As the dietary hypothesis applies for explaining the acquisition of most of dendrobatoid alkaloids, the amount of variation within species and populations correlates with the spatial and temporal variation in the availability of dietary items in the forest (Saporito et al. 2006, 2012), genetic/transcriptomic differences in their resistance (Santos et al. 2016; Tarvin et al. 2017), and metabolic capabilities (Abderemane-Ali et al. 2021; Márquez 2021; Alvarez-Buylla et al. 2022; Jeckel et al. 2022). The first discovered ecological variable that determines chemical variation within dendrobatoids is population (two or more populations from the same species). These studies found that the similarities of alkaloid profiles are correlated with geographic distance and phylogenetic relationships. Interestingly, in some cases where the same species inhabit several locations, they differ in alkaloid composition (Saporito et al. 2006; Mina et al. 2015). In contrast, some different species who live in sympatry share alkaloid profiles (Saporito et al. 2007), while others differ (Myers et al. 1995). The developmental stage also determines differences in alkaloid profiles between juveniles and adults (Daly et al. 2002), and differences between males and females have been proven at least within *O. pumilio* (Saporito et al. 2010). The same heterogenicity on frog’s habitats (e.g., vegetation, temperature, humidity, pluviosity, microbiome) that affects the availability of chemically defended preys, determines that not all arthropods be equally chemically defended (Saporito et al. 2009), probably because they also acquire alkaloids through diet. These complex inter-relation between abiotic factors, habitats, prey items, and frogs could be endangered by deforestation, and probably lead to a decrease in the effectiveness of chemical defences as anti-predatory strategy (Moskowitz et al. 2020).

## Chemical Characterization Overview

Biological questions are the start point to decide the chemical approaches and methods needed to answer those questions. Many improvements have been made for extraction, separation, and identification of natural products since 1963, when the first chemical analysis on skin secretions from dendrobatoids was performed. In the same way, conservation strategies for the preservation of biodiversity have changed over the years modifying the approaches of how scientists have collected, extracted, and analyzed the amazing chemical diversity produced and secreted by different organisms.

### Extraction

There is no such thing as a perfect extraction method that enables the extraction of all metabolites from an organism. Choosing the best extraction method for a specific analysis involves various trade-offs and will depend on the biological question that wants to be answered. When the objective is to obtain a higher yield of pure compounds from the matrix, such as for structure elucidation purposes, a classic approach involving the combination of tissues from multiple specimens and several steps of fractionation/purification is highly desirable. This targeted extraction increases the likelihood of obtaining at least 1 mg of pure compound. On the other hand, if the primary interest lies in analyzing inter-individual differences in broader chemical profiles for a specific biological process, it is important to maintain specimen identity and minimize the number of untargeted extraction steps to avoid loss of trace compounds. These two approaches have been employed in the analysis of dendrobatoids, using seven general extraction methodologies (with some minor modifications) (Table 2).

**Table 2:** Extraction procedures followed for alkaloid extraction of species from the superfamily Dendrobatoidea. GC: Gas chromatography, LC: Liquid chromatography

	Type of sample	Solvent	Number of extraction steps	IV	IS	Reference
Technique 1 (Solvent targeted extraction)	Mixture of skins or less frequently individual whole skins	﻿ Methanol (20-fold excess with weight skin)	1. Maceration in mortar 2. Dilution in H_2_O 3. Extraction with chloroform (3x) 4. Evaporation 5. Dilution in hexane 6. Acidification 7. Basification 8. Extraction with chloroform (3x) 9. Addition of ﻿anhydrous Na_2_SO_4_ 10. Evaporation 11. Reconstitution in methanol * Volumes employed in mL	2 μL (GC)	Not employed	(Daly et al. 1994c)
Technique 2 (Solvent targeted extraction)	Whole individual skin	Methanol (1 mL)	1. Acidification 2. Evaporation 3. Dilution in H_2_O 4. Extraction with hexane (4x) 5. Basification with ﻿NaHCO_3_ 6. Extraction with ethyl acetate (3x) 7. Addition of ﻿anhydrous Na_2_SO_4_ 8. Evaporation 9. Reconstitution in methanol * Volumes employed in μL	1 μL (GC)	﻿10 µg of ((-)-nicotine 99%,	(Saporito et al. 2010)
Technique 3 (Solvent untargeted extraction)	Whole individual frogs	﻿70% ethanol, 80% methanol (totally covering specimens)	1. Evaporation 2. Reconstitution in ﻿chloroform (for lipophilic) or ﻿0.05 M acetic acid (for hydrophilic alkaloids) * Volumes employed in μL	Not specified	Not employed	(Mebs et al. 2014, 2018)
Technique 4 (Solvent untargeted extraction)	Individual dorsal section skins	Methanol (1 mL)	1. Homogenization 2. Evaporation 3. Reconstitution in methanol and vortex 4. Centrifugation and separation * Volumes employed in μL	2 μL (GC) 1 mL (LC)	﻿25 µg of D3-nicotine	(McGugan et al. 2016)
Technique 5 (Solvent untargeted extraction)	Individual dorsal section skins	﻿Ethanol (0.3 mL) after RNA extraction with Trizol	1. DNA precipitation 2. Centrifugation and separation 3. Protein precipitation 4. Centrifugation and separation 5. Evaporation 6. Reconstitution in methanol:chloroform * Volumes employed in mL	5 μL (LC)	﻿1 μM D3-nicotine	(Alvarez-Buylla et al. 2022)
Technique 6 (SPME targeted extraction)	Whole pair of skins	Solvent free procedure	1. Homogenization in liquid nitrogen 2. Transfer to SPME vial and sampling	SPME fiber (GC)	Not employed	(Gonzalez et al. 2021)
Technique 7 (Untargeted MasSpec Pen)	*In vivo*	5% EtOH in water	1. MassSpec Pen tip was gently held in contact with the skin of the frog for < 15 s	NA	Caffeine	(Krieger et al. 2022)

*IV = Injection volume, IS = Internal standard, *Additional note about the order of magnitude of the volumes of solvents employed in the procedure

Between the 1960s and 1990s, between 3000 to 5000 skins were collected from different endemic locations in Central and South America (See details in Table 3 and Online Resource 1). This high amount of skins collected allowed the amount of sample to be sufficient to develop 1H and 13C NMR analysis, and even X-ray diffraction to elucidate structures of lipophilic alkaloids (Daly et al. 1971, 1980, 1988; Tokuyama and Daly 1983; Spande et al. 1999). The extraction protocols included several fractionation and purification steps to isolate pure compounds from the complex frog alkaloid cocktail, which typically consists of 30 or more compounds. Furthermore, as frog skin contains various fatty acids, it was necessary to eliminate these undesired compounds from the extract using hexane. The discovery of the extensive chemodiversity present in poison frogs belonging to the superfamily Dendrobatoidea was a remarkable breakthrough in the field of natural products research. However, the ecological consequences of these extractions, coupled to the illegal traffic that emerged after dendrobatoids became coveted collector's items, are of utmost concern.

**Table 3:** Summarized information about the discovery of families of alkaloids that can be found in dendrobatoids. Extended information about the conditions employed for the structure elucidation and the availability of their chromatograms, mass spectra and mass spectra fragments could be found in Online Resource 1

Alkaloid type	Alkaloids detected	Quantity of Alkaloid	Analytic technique	Ref
Batrachotoxins	BTX, BTXa, pBTX, hBTX	28 mg hBTX, 43 mg BTX + pBTX + BTXa, 39 mg BTXa	HR-MS, NMR, UV, X-ray crystallography	(Tokuyama et al. 1968)
Batrachotoxins	BTX, BTXa, BTXb, BTXc	30 mg	NMR, IR, HR-MS	(Daly et al. 1965)
Batrachotoxins	BTX, hBTX, BTXa, DQH (195B)	500 ug BTX, 300 ug hBTX,	HRMS, GC–MS	(Myers et al. 1978)
Batrachotoxins	BTX, BTXa, pBTX, hBTX	20 ug BTX, 10 ug hBTX, 20 ug pBTX, 30 ug BTXa per skin	TLC, MS(EI), MS(CI), MS(quantitative), GC–MS, GC-FID	(Myers and Daly 1976a)
Histrionicotoxins	HTX 283, isodihydroHTX 285, neodihydrohistrionicotoxin 285, allodihydrohistrionicotoxin 285, isotetradihydrohistrionicotoxin 287, tetrahydrohistrionicotoxin 287, octahydrohistrionicotoxin 291, HTX-D 287, HTX-267, HTX-259, HTX-239, Others: 219–1, 219–2, 223, 231, 235, 237, 239 243, 259, 283, 323 (maybe PTX-B)	190 ug HTX 283, 290 ug isodihydroHTX 285, < 15 ug neodihydrohistrionicotoxin 285, > 15 ug allodihydrohistrionicotoxin 285, < 10 ug isotetradihydrohistrionicotoxin 287, < 5 ug tetrahydrohistrionicotoxin 287, < 10 ug octahydrohistrionicotoxin 291, 40 ug HTX-D 287, < 5 ug HTX-267, < 5 ug HTX-259, < 5 ug HTX-239 per skin	TLC, MS(EI), MS(CI), MS(quantitative), GC–MS, GC-FID	(Myers and Daly 1976a)
Histrionicotoxins	HTX, dihydroisohistrionicotoxin	53 mg HTX, 7 mg dihydroisohistrionicotoxin and 49 mg of other alkaloids	X-ray crystallography	(Daly et al. 1971)
Pumiliotoxins	PTXa (307A, 307A”), PTXb (323A), DQH (195B)	80 ug PTXa, 120 ug PTXb, 0–70 ug PTXc per skin	TLC, MS(EI), MS(CI), MS(quantitative), GC–MS, GC-FID	(Myers and Daly 1976a)
Gephyrotoxins	GTX (called HTX-D)	Not reported	HR-MS, NMR	(Tokuyama et al. 1974)
Gephyrotoxins	Gephyrotoxin and dehydrogephyrotoxin	Not reported	NMR, HR-MS, X-ray crystallography	(Daly et al. 1977)
Pumiliotoxins	PTXa (307A, 307A”), PTXb (323A)	1.5 mg PTXa, 1.5 mg PTXb	UV, NMR, IR, HR-MS	(Myers and Daly 1976b)
Pumiliotoxins	PTX 251D, PTX 307A, PTX 323A	Not reported	X-ray crystallography, NMR	(Daly and Myers 1967)
Pumiliotoxins	PTX 251D, PTX 307A’, PTX 307A” PTX 323A, alloPTX 267A, alloPTX 323B’, alloPTX 323B”, alloPTX 339A, alloPTX 339B	Not reported	NMR, IR	(Daly et al. 1980)
Pumiliotoxins	237A, 253, PTX-B(323-A), an isomer of PTX-B(323-B), 237B, 265 (degradation artifact?), 281A	115 ug of 237A, 34 ug of 253, 8 ug of 323A, 3 ug of 323B, per 100 mg of skin	MS(CI), MS(EI), GC–MS	(Tokuyama et al. 1984)
Allopumiliotoxins	alloPTX 253, alloPTX 267A, alloPTX 297A, alloPTX 323B	Not reported	NMR, HR-MS	(Daly et al. 1980)
Homopumiliotoxins	hPTX 223G	8 mg	HRMS, GC–MS, NMR	(Tokuyama et al. 1987)
Decahydroquinolines	DQH (195B)	17 mg PTXa, 20 mg PTXb, 16 mg PTXc	HR-MS, NMR, X-ray crystallography	(Daly et al. 1969)
Pyrrolizidines	223B, 223H, 237G, 251 K	Not reported	GC–MS(EI), GC–FTIR	(Garraffo et al. 1993)
Indolizidines	223AB, 239AB	Not reported	GC–MS(CI), GC–MS(EI), HR-MS	(Daly et al. 1978)
Indolizidines	223AB	Not reported	GC–MS and NMR of synthetic diastereoisomers	(Spande et al. 1981)
2,5-disubstituted pyrrolidines	197B	Not reported	GC–MS(CI-NH3), GC–MS(CI-ND3), GC-FID, GC-HRMS, NMR	(Daly et al. 1986)
Lehmizidines	275	Not reported	GC–MS(CI-NH3), GC–MS(CI-ND3), GC-FID, GC-HRMS, NMR	(Daly et al. 1986)
2,6-disubstituted piperidines	225B	Not reported	GC–MS(CI-NH3), GC–MS(CI-ND3), GC-FID, GC-HRMS, NMR	(Daly et al. 1986)
2,6-disubstituted piperidines	241D	Not reported	GC–MS, NMR	(Edwards et al. 1988)
Quinolizidines	195C	Not reported	HR-MS, GC–MS(CI-NH3), GC–MS(EI), GC–FTIR	(Jones and Gorman 1999)
Epibatidines	208/210 Epibatidine	60 mg	HR-MS, NMR, GC–FTIR	(Spande et al. 1992)
Tetrodotoxins	TTX	Not reported	NMR, IR, TLC	(Mosher et al. 1964)
Tetrodotoxins	TTX, 4-epiTTX, 4,9-anhydroTTX	Not reported	HPLC-FLD, TLC	(Daly et al. 1994a)

The charismatic appearance of dendrobatoids and their pharmacological properties put them in the spotlight and nowadays several of these species are endangered or closed to extinction, as *Oophaga lehmanni* (Betancourth-Cundar et al. 2020). Conservation efforts are even more important now with amphibians facing chytridiomycosis panzootic (Scheele et al. 2019) and with special importance in the northern Andean countries, as Colombia, that possess both the highest species diversity and the highest diversity of at-risk dendrobatoid species (Guillory et al. 2019). The greater awareness of conservation comes also with an effort for diminishing the amount of organic solvents employed in the extraction procedures. For these reasons, only after structure elucidation was accomplished by Daly et al., and improvements in the sensitivity of instruments was possible, technique 1, big mixtures of dendrobatoid skins extracted with big volumes of organic solvents for alkaloid fractionation (Daly et al. 1994c) was replaced by technique 2: a small number of skins fractionated individually with small amounts of organic solvents (Saporito et al. 2010) (Table 2). Then, other researchers have the opportunity to diminish the production of waste residuals avoiding alkaloid fractionation step, as their focus lies not on isolating individual compounds. (Mebs et al. 2014, 2018). More recently researchers opt for collecting the minimal number of skins needed to compare chemical profiles to test an ecological/chemical hypothesis without losing any trace compounds performing alkaloid fractionation (McGugan et al. 2016) (Table 2). Recently, the application of another solvent-free extraction procedure employing SPME fibers has been applied for the extraction of alkaloids and VOCs in *Silverstoneia punctiventris*, and this could be applied for sampling other species (Gonzalez et al. 2021) (Table 2). Making greater conservationist efforts, some non-lethal methods have also been explored. Small methanol-laced Kim-wipes (Kymberly-clark, Roswell, GA, USA) placed over the frog’s skin (Clark et al. 2006), cotton swabs (Cardall et al. 2004; Mebs and Pogoda 2005) or small pieces of filter paper (Schulte et al. 2017) with subsequent solvent-desorption. Even electrical stimulation has been employed to promote alkaloid secretion (Clark et al. 2006; Hantak et al. 2013; Schulte et al. 2017). Non-lethal methods have not been broadly applied because there is contradictory evidence regarding their efficacy with lipophilic alkaloids extraction. Fortunately, Krieger et al. (2022) recently demonstrated that* in vivo* methods could be applied to perform targeted and untargeted analysis of alkaloids in dendrobatoids using the MassSpec Pen (Krieger et al. 2022). Hopefully, in the coming years, the current gold standard method lethal for performing chemical analysis from dendrobatoids (that compromised frog skinning and posterior solvent extraction) will be replaced with more *in vivo* alternatives. However, one of the most significant challenges in comprehending the diverse array of functions exhibited by metabolites found in dendrobatids is the limited availability of pure standards. Since these standards are primarily restricted to dendrobatoids (and their dietary sources), and organic synthesis can be extremely challenging in some cases, novel methodologies for purifying them are still required. Once we are able to obtain purified compounds, we can conduct bioassays to investigate their functions (e.g. toxicity, unpalatability, repellency, antimicrobial activity) or potential synergistic effects in chemical communication. The challenge in this case is to obtain a sufficiently high quantity of pure compounds that enables the performance of these experiments, or to perform NMR and X-ray diffraction experiments to complete structure elucidation.

### Separation

**Fig. 4:**
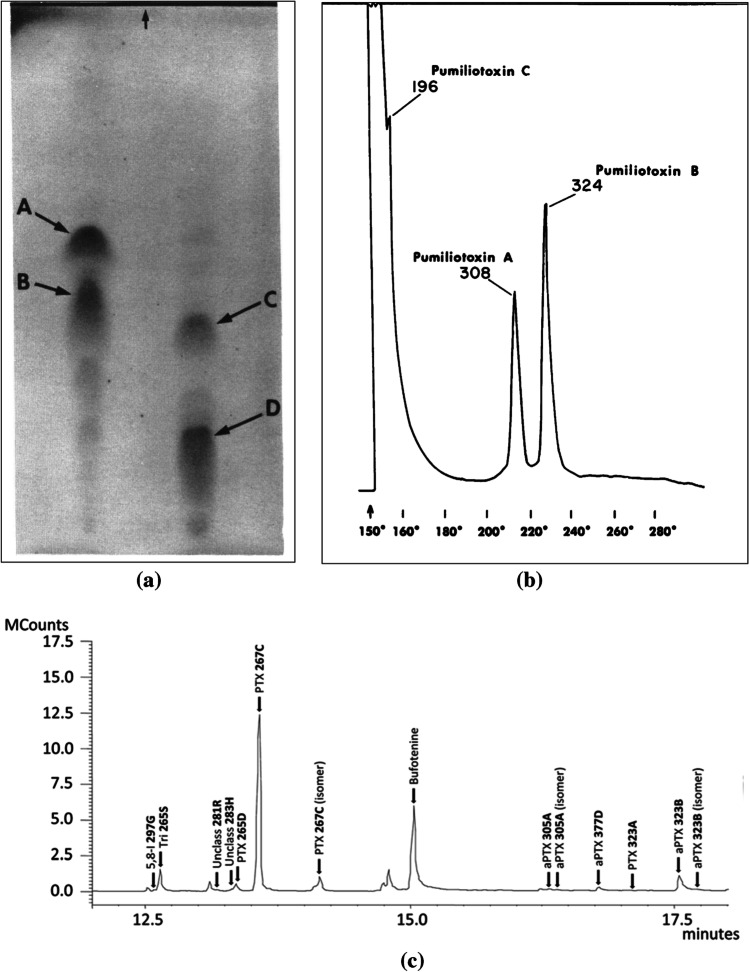
Examples of separation techniques used for chemical analysis from species of Dendrobatoidea superfamily. (**a**) Representative thin-layer chromato-plate of alkaloids from *Oophaga histrionica* (Guayacana population) on left, and *Oophaga pumilio* (Bastimentos population) on right. (Arrows and letters A, B, C and D, describe the separation of the following alkaloids: **283A** (histrionicotoxin), **285A** (isodihydrohistrionicotoxi), **307A** (pumiliotoxin A), **323A** (pumiliotoxin B). Reproduced from Copyright © 1976, (Myers and Daly 1976b); (**b**) Gas chromatogram of alkaloids from a population sample of *Dendrobates pumilio* (10 frogs from Isla Bastimentos, Panama). Copyright © 1976, Reproduced from (Myers and Daly 1976b); (**c**) Gas chromatograms of alkaloid profiles of individual specimens of the toad *﻿Melanophryniscus moreirae*. Copyright © 2015, (Jeckel et al. 2015)

First, thin layer chromatography (TLC) was used for preliminary screening amphibian lipophilic alkaloids (Märki and Witkop 1963), when there was little knowledge about the diversity of alkaloids contained in dendrobatoids’ skin (Fig. 4A). Then in 1986, gas chromatography, which has great sensitivity and reproducibility, became the main technique (Daly et al. 1986). A standard heating program starting at 100 °C and rising until 280 °C, was employed for the separation, but during the ‘60 s most of the peaks were poorly resolved (Fig. 4B), probably because of the technology available for the fabrication of column’s stationary phase. Initial packed columns (as OV-1) were replaced by wall-coated, open tubular (WCOT) or capillary columns (Sciarrone et al. 2015). Years later, with these improvements, using the same temperature program, at a rate of 10 °C per min, better resolution was achieved and extended to the analysis of other alkaloid-containing amphibians (Fig. 4C). Since that moment retention times (Rt) were registered for each alkaloid and used as a reference for proposing possible identifications. An alternative heating method, aimed to incorporate more volatile compounds, starts at 40 °C and rise to 6 °C/min until 300 °C (Gonzalez et al. 2021).

Some higher molecular weight lipophilic alkaloids were analyzed by Daly et al. (2005) using high performance liquid chromatography (HPLC) (Daly et al. 2005), and even allowed the fractionation/isolation of some of them (Fitch et al. 2003; Mortari et al. 2004). More recently, LC–MS has been also employed for the analysis of alkaloids (McGugan et al. 2016; Shiraishi et al. 2017; Fischer et al. 2019; Protti-Sánchez et al. 2019; Martin et al. 2020; Moskowitz et al. 2020) and proteins (Caty et al. 2019; O’Connell et al. 2021) in dendrobatoids employing a reversed-phase gradient method.

Chemical analysis on some dendrobatoid species has failed to unequivocally detect lipophilic alkaloids employing TLC separation (Santos and Cannatella 2011), and actually discovered the presence of such alkaloids using GC separation, as the case of *Epipedobates boulengeri* (Cipriani and Rivera 2009). For many years, this species was erroneously used as a reference for absence of alkaloids, based on TLC analysis. For this reason, it is recommended to avoid using coarse techniques as TLC for the chemical analysis of this group of amphibians, and to employ GC or LC instead.

For the analysis of hydrophilic alkaloids, TLC was also used for preliminary screening (Mosher et al. 1964), but LC became the most commonly used type of chromatography because TTXs have high temperature stability, low volatility and low solubility in organic solvents (Daly et al. 1994a; Ibáñez and Smith 1995; Pires et al. 2005). GC has also been employed for the analysis of non-amphibian organisms, but it requires derivatization of the TTX and its analogous structures prior to analysis (Man et al. 2010).

### Characterization

Many techniques have been used for molecular characterization of the metabolites derived from dendrobatoids. MS is the most informative and transversal technique applied to many species and compounds previously separated by GC, LC or other types of chromatography. Mass spectrometers could be classified in low- and high-resolution instruments. High-resolution mass spectrometers include time of flight (TOF), orbitrap, and Fourier transform ion cyclotron resonance (FTICR) mass analyzers. Their ability to distinguish ions of different elemental composition is determined by mass resolution, that depends on the instrument resolving power. High resolution instruments should have a resolving power (m/Δm50%) > 10, 000. Consequently, high mass accuracy (rms) is obtained, and trough the analysis of isotopic distributions the prediction of elemental compositions of metabolites is facilitated (Xian et al. 2012). Low-resolution mass spectrometers include single quadrupole, triple quadrupole, and orthogonal acceleration quadrupole ion trap mass analyzers.

We have separated GC and LC characterization techniques applied to the analysis of lipophilic and hydrophilic amphibian alkaloids.

Lipophilic alkaloids separated by GC have been analyzed with electron ionization-mass spectrometry (EI-MS) in single quadrupole and TOF analyzers. During the elucidation process of alkaloid structures, many empirical formulas were obtained by high-resolution mass spectrometry and molecular masses were confirmed by ammonia chemical ionization-mass spectrometry (CI-MS) (Daly et al. 2005). Hydrogen deuterium exchange (HDX), which provide complementary information about which functional groups may be present in metabolites (Lam and Ramanathan 2002) has been applied employing ND_3_ in place of NH_3_ in CI experiments (Daly et al. 2005). One limitation for accomplishing level 1 metabolite annotations and quantitative measurements in MS is the lack of analytical standards to estimate the relationship between the quantity and the signal for each analyte. Because of this, MS measurements should be considered differential rather than quantitative (Daly et al. 2005). Unfortunately, only a couple of lipophilic dendrobatoid alkaloids are commercially available by vendors, such as batrachotoxin and epibatidine (Shiraishi et al. 2017) (See Section "Selecting a separation and characterization by GC–MS or LC–MS"). Most of the lipophilic alkaloids from dendrobatoids have been usually characterized by GC–MS, but other lipophilic alkaloids with high molecular weight (~ > 450 uma), such as batrachotoxins, could be analyzed exclusively by direct injection or by LC–MS (Dumbacher et al. 2000; Protti-Sánchez et al. 2019).

Lipophilic alkaloids were either separated by HPLC and analyzed by tandem mass spectrometry (MS/MS) for obtaining their fragmentation spectra, or by direct injection (when isolation was achieved by other types of chromatography) employing high resolution (HR) spectrometers. Even though most of the analyses were performed using electrospray ionization (ESI) as interface, atmospheric pressure chemical ionization (APCI) employing D_2_O in place of H_2_O has been useful for determining the number of exchangeable hydrogens (Daly et al. 2005). Recently, Jeckel et al. (2020) have also used desorption electrospray ionization mass spectrometry imaging (DESI-MSI) to visualize spatial distribution of alkaloids on frog tissues (Jeckel et al. 2020).

For the analysis of hydrophilic alkaloids, specifically tetrodotoxin and its analogues, HPLC–MS/MS and high-performance liquid chromatography coupled to fluorescence detector (HPLC-FLD) have been the main techniques. HPLC-FLD was first used for quantitative comparisons (Yasumoto and Michishita 1985), but when it was discovered that TTX and its analogous structures have large differences in their fluorescence intensities (Asakawa et al. 2012), fewer and fewer researchers used this detector when high precision in quantification was required. Nevertheless, it could be useful for tracking tetrodotoxins in a frog’s skin extract (Mebs et al. 2018). HPLC–MS has the advantage to help overcome the differential fluorescence intensities, allowing for a more accurate quantification using different analytical standards of tetrodotoxins (Mahmud et al. 1999; Horie et al. 2002).

Other techniques applied to the characterization of lipophilic alkaloids, include gas chromatography coupled to flame ionization detector (GC-FID) were most of the quantitative analysis have been made (Daly et al. 2005). Also, gas chromatography coupled to Fourier transformed infrared spectroscopy (GC–FTIR) has been extremely useful to provide structural insights into functional groups and stereochemical configurations of many alkaloids (Daly et al. 2005; Jeckel et al. 2022). Table 3 is a condensed version of Online Resource 1 where the identity of the alkaloids and publications that conducted to the discovery of different families of alkaloids found in dendrobatoids is summarized.

## Eco-Metabolomics Workflow

**Fig. 5:**
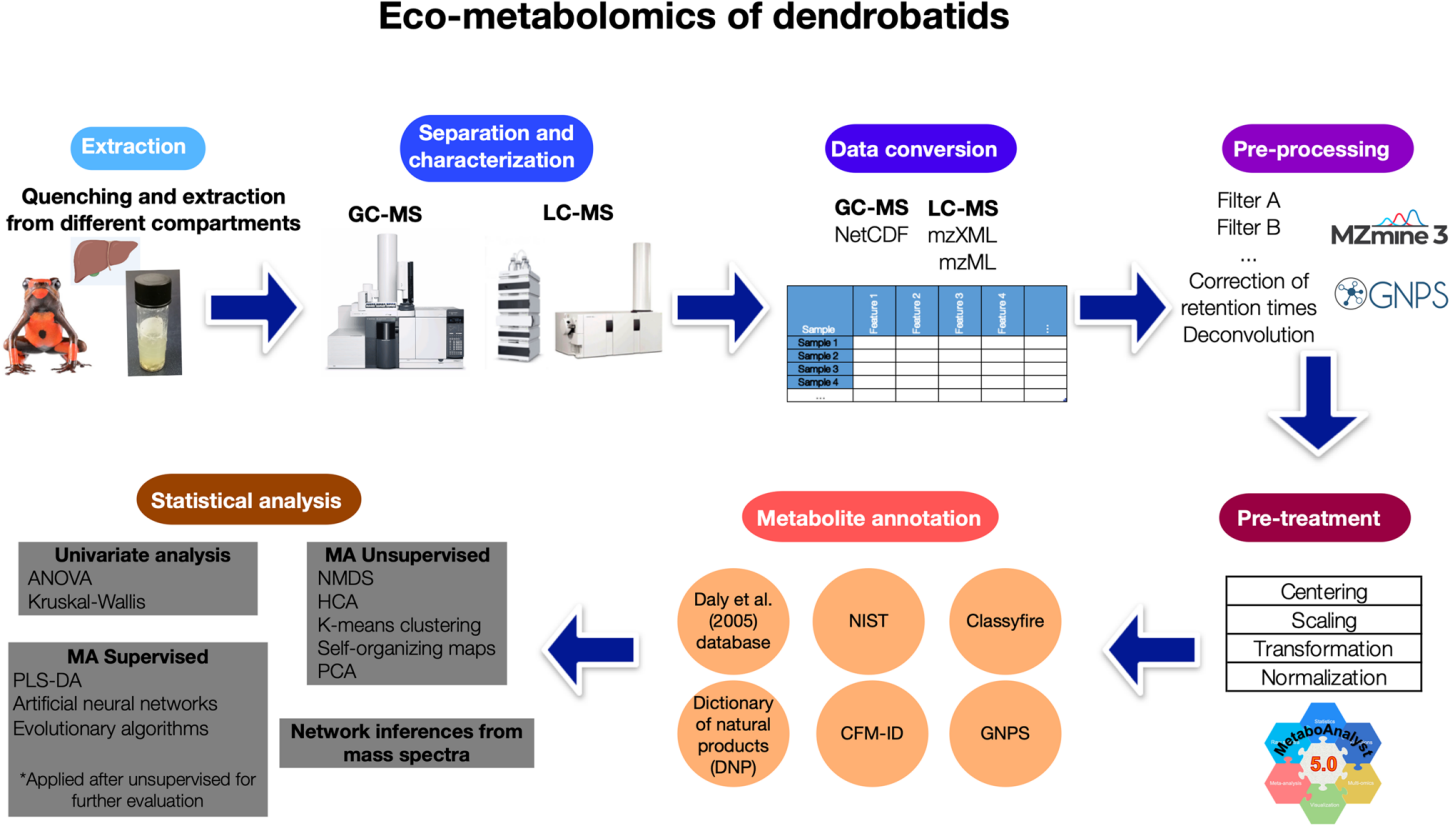
Eco-metabolomics workflow suggested for data-analysis from GC–MS and LC–MS analyses

The general Eco-metabolomics workflow suggested for data-analysis from GC and LC analyses coupled to mass spectrometry is summarized in Fig. 5. Employing this workflow, we hope that the following steps could be used as a guidance to update the chemical analysis of dendrobatoids to the current Metabolomics capabilities. In addition, we hope that sharing raw files from Eco-metabolomics studies derived from dendrobatoids in the following years it will become mandatory, in an analogue form as sharing DNA-sequences is now mandatory in Genomics.

### Extraction Methods for Metabolomic Analysis

Most of the extraction methods employed in the chemical analysis of dendrobatoids involved solvent-extraction and alkaloid fractionation (See Section "Extraction"). The lack of comparisons after implementing different solvents, extraction steps or quenching methods makes it hard to decide which protocol offers better extraction efficiency for a specific group of compounds. In addition, the thermal and pH stability of most the compounds is unknown. On the top of that, due to the low availability of analytical standards from the different alkaloid families (see Section "Quantification and semi-quantification") there are not studies available evaluating the matrix effects of different extraction procedures.

#### *In Vivo* Methods and Euthanasia Protocols

The extraction efficiency of *in vivo* methods should be properly estimated in the future with the aim to replace the whole-skin-extraction with the analysis of dendrobatoid secretions employing mild electrical stimulation. Currently, despite non-lethal methods have been implemented (Clark et al. 2006; Hantak et al. 2013; Schulte et al. 2017; Krieger et al. 2022) there are many doubts about its efficiency. Solvent or thermal extraction from sorbent materials such as swabs, PDMS patches, filter paper, or methanol-laced Kim-wipes, could be implemented to survey the chemical profiles of different species directly on the field, taking advantage of the increased sensitivity of high-resolution mass spectrometers such as TOF, Orbitrap and Cyclotron (Aksenov et al. 2017).

Even for the whole-skin-extraction there is also a lack of consensus method of euthanasia for the chemical analysis on dendrobatoids. Most of studies before the decade of 2010’s does not provide details about methods of euthanasia, but immersion in ethanol (Saporito and Grant 2018) was a common method employed in the herpetological community. Anesthetics provided by injection ﻿(Mebs et al. 2014) or orally (Amézquita et al. 2017) has been also employed, but conducted to a rapid incorporation into the amphibian skin (Saporito and Grant 2018) that could hinder chromatographic and toxicity analyses. Topical anesthetics in the ventral skin surface such as benzocaine (McGugan et al. 2016; Caty et al. 2019; Protti-Sánchez et al. 2019; Alvarez-Buylla et al. 2022) has been also applied, sometimes followed by ﻿cervical transection to complete euthanasia. Washing the body with distilled water to avoid the interference of the anesthetic in the chromatographic profile was proven to be effective at least in one study (Protti-Sánchez et al. 2019). Gradual cooling following immersion in liquid nitrogen has been also employed with dendrobatoids (Gonzalez et al. 2021; Jeckel et al. 2022) and other amphibians (Brunetti et al. 2015). Currently, it is necessary to provide detailed euthanasia protocols to ﻿University’s Institutional Animal Care and Use Committee (IACUC) but is uncertain how different protocols influences the extraction of different metabolites.

#### Extraction in Tissues or Compartments

Skin has been the main compartment used for the chemical analysis in dendrobatoids. However, it has been proven that alkaloids have been detected in other tissues such as oral mucous, stomach, liver, intestines, kidney, muscle, oocytes, eggs (Stynoski et al. 2014; O’Connell et al. 2021; Alvarez-Buylla et al. 2022; Jeckel et al. 2022) and blood has been collected to perform proteomic analysis (Caty et al. 2019). Depending on the study, samples from these other compartments have been collected directly in a solvent, flash frozen (Caty et al. 2019; O’Connell et al. 2021) or previously dried at 60ºC (Jeckel et al. 2022).

#### Quenching and Extraction

In general, for the extraction of most natural products it is advisable to collect fresh samples and applied a quick quenching method to inactivate degradation reactions. Most of the studies have employed immersion of the tissue in methanol or other solvent as quenching/extraction method (Daly et al. 1994c; Saporito et al. 2010; Mebs et al. 2014, 2018; McGugan et al. 2016; Alvarez-Buylla et al. 2022). A broadly used and effective quenching method for the extraction of other natural products has been flash-freezing in liquid nitrogen (Mushtaq et al. 2014; Bonat Celli et al. 2016), but this methos has been less frequently employed in dendrobatoids (O’Connell et al. 2021; Jeckel et al. 2022). Liquid nitrogen offers the advantage that could be applied for a double purpose in amphibians: euthanasia and quenching, and even subsequently facilitate sample homogenization with mortar and pestle. It has been proven that amphibian skin peptides could suffer partial or total degradation in the absence of a a proper quenching method (Samgina et al. 2016), so it is advisable to consider methanol, or methanol/water as extraction solvents (Samgina et al. 2018), or water with snap-freezing (Jiang et al. 2014) followed by lyophilization for thermal sensitive compounds.

For untargeted metabolomics (see Section "Selecting a separation and characterization by GC–MS or LC–MS") usually mixtures of methanol and water are the most popular combinations because this broader polarity extracts a wide range of metabolites, such as sugars, amino acids, organic acids, alkaloids and phenolic compounds (Mushtaq et al. 2014). However, generally water is non-compatible with GC–MS injection for its high large vapor volume in the injector, so solvent selection should be compatible with the polarity of the metabolites of interest, their solubility and the chromatographic platform that will be employed later. Sample treatments such as solid phase extraction (SPE), centrifugation and filtration are highly recommended because this would extend the life of the stationary phase of chromatographic columns and possible decrease ion suppression effects (Alseekh et al. 2021). Based on the principles of green chemistry, microextraction techniques such as head space solid microextraction ﻿(HS-SPME) recently has been successfully applied to analyze volatile compounds and alkaloids in dendrobatoids (Gonzalez et al. 2021) facilitating the analysis of amphibian secretions with a solvent free method and using less steps. Other microextraction techniques as single drop microextraction (SDME), dispersive liquid–liquid-microextraction (DLLME) and hollow-fiber liquid-phase microextraction (HF-LPME), combined with green solvents (Carasek et al. 2021) need to be implemented in the following years for the chemical analysis of dendrobatoids.

### Selecting a Separation and Characterization by GC–MS or LC–MS

The first decision that needs to be made is whether the analysis will be targeted or untargeted. This decision will depend on the specific question that needs to be answered. The targeted analysis, usually accompanied by selective extraction procedures usually seeks an accurate quantification of specific metabolites with a previously known structure and that represent a specific pathway(s) or class(es) of molecules. This procedure requires internal standards and needs that certain conditions be optimized/tune in the chromatographic separation and mass spectrometer to maximize the detection of target molecules (Roberts et al. 2012). On the other side, untargeted analyses seek to trace all the metabolites that can be possibly detected in a sample, including identified and unidentified compounds, because the main objective is to make relative estimations and comparison between samples or groups. This procedure will lead to the detection of hundreds or thousands of molecular features (peaks with specific retention time and mass to charge ratio *m/z*) that do not directly reflect the metabolite identity, because just a portion of these features will be annotated (Liu and Locasale 2017).

Most of the studies made have performed a targeted analysis of extracts enriched in alkaloids. As the purpose of this review is to motivate other researcher to extend the spectrum of chemical analysis performed in dendrobatoids, when the main question is related to compare chemical profiles, we encourage to implement untargeted approaches for the analysis of proteins, peptides, biogenic ammines, volatile organic compounds (VOCs) and alkaloids. However, when the goal of the study is to investigate or isolate a specific compound or class of compounds, a targeted approach would be more appropriate. GC–MS and HPLC–MS/MS instruments offer different advantages and disadvantages and selecting one of these platforms will depend on the metabolites of interest.

Metabolites analyzed by GC–MS need to have masses lower than 400–500 Da (Liu and Locasale 2017; Carazzone et al. 2021), which make the technique suitable for gases or VOCs, but not for peptides, proteins, or thermo-labile small molecules. This platform offers highly reproducible retention times and has well developed mass spectral libraries to annotate compounds. Some of the compounds, such as highly polar metabolites will require derivatization which could lead to undesired byproducts. Most of the alkaloid profiles described in dendrobatoids have been made on this platform and some reference mass spectra of dendrobatoid alkaloids are represented in GC–MS libraries (see Section "Data derived from GC–MS").

LC–MS offers a higher versatility to analyze a broader spectrum of molecules with different polarities and masses that can be ionized by themselves or by addition of acids. In contrast, the retention times and separation conditions are less reproducible than by GC–MS, as well as the mass spectral libraries are less developed, which makes annotation even more challenging (Liu and Locasale 2017). Some targeted analysis specific for batrachotoxin and tetrodotoxin has been made in dendrobatoids employing LC–MS/MS, because GC–MS is not appropriate for the analysis of these two alkaloids (see Section "Data derived from GC–MS").

Following chromatographic separation and mass spectrometry analysis, the large amount of data generated needs to be processed following a standardized procedure that include data conversion, pre-processing, pre-treatment, metabolite annotation, univariate statistics, multivariate statistics, network inference and sharing data in public repositories. This last step currently is not a common practice for chemical profiles from dendrobatoids, but we hope that this document motivates researchers to share their data in open repositories after performing targeted and untargeted analyses. We hope that chemical profiles from studies already publish since 1960’s be also uploaded on these repositories and prove their value to perform meta-analyses of dendrobatoid chemodiversity, including annotated and non-annotated compounds.

### Data Conversion

One of the most commons raw data formats available for GC–MS runs are.D (Aglient instrument),.lcd (Shimadzu instrument) and.raw (Thermo Fisher instrument). Conversion to ﻿open-source formats usually supported by many software packages as.cdf,.mzXML or.mzML could be performed using some of the tools available in the vendor software, but ProteoWizard (also called MSconvert) (https://proteowizard.sourceforge.io/download.html) is an open-source free tool able to convert formats from all vendors. During file conversion it is important to choose Peak Picking with Vendor checked in the Filters section, to centroid the data. Then, indicate MS-Levels 1–2 and click “Add” to correctly add the filter.

Following the principles of transparency in Metabolomics it is recommended to share raw data and downstream results in repository databases. Some examples includes MetaboLights (Haug et al. 2013) (https://www.ebi.ac.uk/metabolights/), the Metabolomics Workbench (Sud et al. 2016) (https://www.metabolomicsworkbench.org/), and GNPS-MassIVE (Wang et al. 2016) (https://massive.ucsd.edu/ProteoSAFe/static/massive.jsp). Equally important, it is essential that following these principles researchers share details about chromatographic conditions, experiments and conditions used in the mass spectrometers, report metabolites using a unified format, include international metabolite identifiers such as CAS number and IUPAC names, and provide detailed metadata associated with the files uploaded into these repositories (Alseekh et al. 2021). ReDu template (https://docs.google.com/spreadsheets/d/1v71bnUd8fiXX51zuZIUAvYETWmpwFQj-M3mu4CNsHBU/edit#gid=450198104, in.tsv format) contain metadata of general interest ﻿for MS-based metabolomics and allows ﻿a subsequent reanalysis with/without other public datasets in the GNPS ecosystem (Jarmusch et al. 2020).

### Pre-Processing

This step aims to use different filters to recognize signal from noise and to facilitate comparisons of the metabolite profiles among samples. Some filters of signal intensities, mass ranges or peak areas could be applied employing vendor software or excel. However, to establish quantitative procedures for discarding less reliable signals there are many Automated Data Analysis Pipelines available to facilitate pre-processing of targeted and untargeted mass spectrometry-based metabolomics data. Peak detection filters and spectral deconvolution comprises the main pre-processing steps. Filters could be applied to exclude molecular features with intensities close to the noise signal or with low prevalence in a group of samples. On the other hand, deconvolution use different algorithms to detect analytes by combining similar peaks into clusters and using their intensities to construct fragmentation mass spectra and align them among samples. Some filters in the pre-treatment could be employed to target the compounds of interest (e.g. compounds with high intensity or with an specific elution time). There are several methods and software packages to perform data pre-processing (see details in (Alseekh et al. 2021)), but MZmine (Pluskal et al. 2010) (http://mzmine.github.io/), ADAP/MZmine (Smirnov et al. 2019) and GNPS (Wang et al. 2016; Aksenov et al. 2017; Nothias et al. 2020) (https://gnps.ucsd.edu/ProteoSAFe/static/gnps-splash.jsp) are some of the tools that offer higher versatility to perform further data pre-treatment, metabolite annotation/visualization and even exploratory statistical analysis.

### Pre-Treatment

Pre-treatments methods ﻿include centering, scaling, transformation, normalization and batch effect treatments. The selection of the most appropriate methods require to check the hypothesis to be tested, the statistical behavior of the molecular feature matrix, and if data fits for a specific treatment (Carazzone et al. 2021). One of the most widely used platforms in the Metabolomics community is Metaboanalyst (Pang et al. 2022) (https://www.metaboanalyst.ca/). This tool is probably the most powerful tool to perform data pre-treatment and statistical analysis. Recently, in their latest version they launch a tool that allows raw data processing for LC-MS1 data (Pang et al. 2021). However, as Metaboanalyst does not support processing spectra from raw GC–MS or LC–MS/MS, it is not generally recommended for data pre-processing or metabolite annotation.

Usage of the pre-processed matrix analyzed in other software packages needs that the file be exported in a separate.CSV file including one of the metadata specified as row/ column before being imported into Metaboanalyst. Once the dataset is uploaded, three conditions must be complaint by the dataset: (1) the sample and variable names must be unique and contain no special characters (e.g., Greek letters); (2) each group from the metadata needs to contain at least three replicates; (3) all data values must be formatted as numeric, except phenotype labels in the metadata. Missing values are allowed and should be indicated as a blank or marked as NA (without quotes). For paired analysis, MetaboAnalyst also checks if the data pairs conform to the specified format. On this platform critic pre-treatments parameters include estimation of missing values, different filtering strategies, and the possibility of normalizing, transforming, and scaling the data using different methods. The Data Normalization Result page show a graphical summary of the data before and after the normalization procedure to validate whether it delivers the desired results. Internal standards can be also recognized by Metaboanalyst to execute normalization. For more details about loading data and performing pre-treatment in Metaboanalyst check (Xia and Wishart 2016). For more details about how to choose the best method for ﻿centering, scaling, and transforming a dataset check (Van den Berg et al. 2006). It is crucial that before applying pre-treatment methods, researchers check if data is fit for analysis and if is relevant for the hypothesis researchers want to test.

### Challenging Metabolite Annotation

More than 40 years of publications on amphibian alkaloids have allowed the creation of an extended database for amphibian lipophilic alkaloids where the spectroscopic characteristics and chromatographic parameters after a GC–MS analysis of almost 800 alkaloids are summarized. From this, more than 500 alkaloids belong to dendrobatoids. This information is found as text files of supplementary information in the publications by Daly et al. and Garraffo et al. (Daly 2003; Daly et al. 2005, 2009; Garraffo et al. 2012). For some alkaloids, their spectral properties are contained within NIST database (https://www.mswil.com/software/spectral-libraries-and-databases/nist20/) and Wiley database (https://www.mswil.com/software/spectral-libraries-and-databases/%20wiley-spectral-libraries/wiley-gcms-libraries/), but there is no reference for linear retention indices (RI) for most cases. The analogue is the retention time (Rt) value from the temperature program employed by Daly et al. (Daly et al. 1994b), but retention times by itself are not reproducible across instruments and laboratories (Strehmel et al. 2008). Thus, despite advances made regarding extraction and separation techniques, the annotation process for dendrobatoid metabolites needs to be updated for the cutting-edge advances made in general Metabolomics.

Annotation and identification levels for metabolites have been defined by the Chemical Analysis Working Group of the Metabolomics Standards Initiative (MSI). Level 1 refers to identified compounds, level 2 is used for putatively annotated compounds, level 3 is used for putatively characterized compound classes, and level 4 is used for unidentified or unclassified metabolites that still can be differentiated and quantified based upon the information contained in the mass spectra. Most of the studies made about the chemical ecology of dendrobatoids have level 2 annotations, and the non-annotated structures have been mainly disregarded. Level 1 identification usually are not possible, because of the lack of commercially available chemical standards of most of dendrobatoid’s alkaloids. However, dark matter, also called “unknown unknowns”, represents the majority of metabolites analyzed in a metabolomics experiment, because instruments collect much more information than it is currently possible to annotate (Da Silva et al. 2015). Current publications that exclude unknown structures that could not be completely elucidated, are probably disregarding a high amount of ecologically and maybe pharmacological relevant information about dendobatids’ “unknown unknowns”.

#### Data Derived from GC–MS

**Fig. 6:**
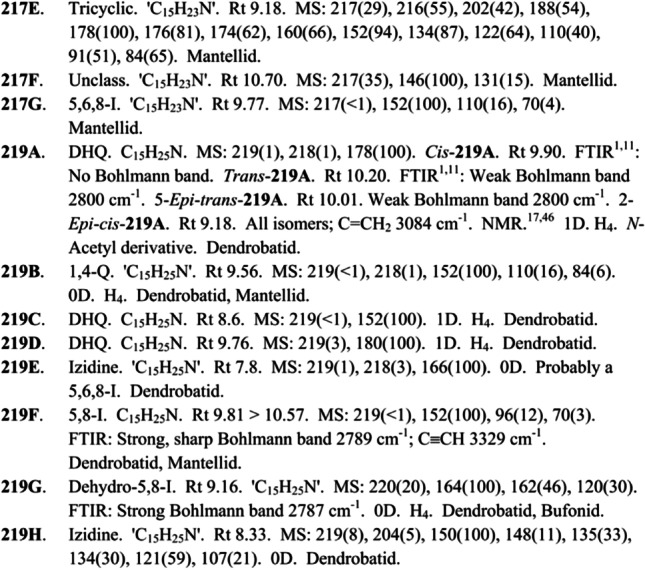
Section from amphibian alkaloid database created by Daly et al. (Daly et al. 2005). Reprinted (adapted) with permission from Daly et al. 2005. Alkaloids from Amphibian skin: A tabulation of over eight-hundred compounds. J Nat Prod 68:1556–1575. doi:10.1021/np0580560 . Copyright © 2005 American Chemical Society

Since 1987, the content of Daly database for GC–MS analysis (Daly et al. 2005) organizes each alkaloid by the code name (bold-faced number + bold-faced letter) ranging from the lower nominal mass to the higher. The letter is organized alphabetically to differentiate individual alkaloids with the same nominal mass (Daly et al. 1987). For example, so far seven alkaloids with a mass of 219 Da, called **219A**, **219B**, **219C**, **219D**, **219E**, **219F**, **219G** and **219H** have been reported. Together with the code name of each alkaloid, the database contained the alkaloid family to which it belongs, its molecular formula, retention time (Rt), diagnostic mass spectral ions, and their respective abundances. If there is information regarding the Rt of different isomers or if they differ in fragmentation patterns, this information is also included. Finally, their occurrence in Dendrobatoid, Bufonid, Mantellid, Myobatrachid anurans, arthropods or plants was specified. For some alkaloids, when FTIR, NMR, CI (by GC or HPLC) analysis are available other features are detailed. Figure 6 shows a small section from this database. Here, three isomers with nominal mass 217 are specified as well as eight isomers with nominal mass 219.

The classical procedure for amphibian alkaloids identification processed in GC–MS analysis, include a first comparison between experimental mass spectra with NIST and Daly databases. For manual comparison, a set of characteristic ions for each alkaloid family usually helps to narrow down the possible candidate structures (Table 4). Then, for each family, other fragments need to be reviewed manually and the coherence with the retention times contained into databases assessed. Garraffo et al (2012) (Garraffo et al. 2012) propose a linear correction of theoretical and experimental retention times, to overcome the lack of linear retention indices (RI) and this method has been employed for Jeckel et al. (2019) (Jeckel et al. 2019). Annotation usually is especially challenging for those families that contain a high number of alkaloids such as indolizidines, izidines and tricyclics, because of the similarity of the mass spectra and the absence of molecular ions after the GC–MS analysis.

**Table 4:** Total number of lipophilic alkaloid families found in amphibian skins and chemical features useful for annotation process. Chemical features obtained from (Daly et al. 1993, 2005, 2009; Saporito et al. 2012)

Class Subclass	# *	Type of alkaloid	Lower molecular mass (m/z)	Higher molecular mass (m/z)	Principal ions by MS
1,4- disubstituted quinolizidines	22	Bicyclic	207	295	110 and 84
3,5-disubstituted indolizidines	23	Bicyclic	167	275	124
3,5- disubstituted pyrrolizidines	23	Bicyclic	167	267	Variable
4,6- disubstituted quinolizidines	6	Bicyclic	195	279	Variable
5,6,8- trisubstituted indolizidines	76	Bicyclic	193	353	70 and 110 or 124 or 138…
5,8- disubstituted indolizidines	77	Bicyclic	167	297	138 or 152 or 180 or 96
5,8- disubstituted dehydroindolizidines	32	Bicyclic	179	275	120 and 136 and 134 or 150 and 148 or 164 or 162
Decahydroquinolines	32	Bicyclic	181	293	152 or 168 or 341
Decahydroquinolines dimers	6	Bicyclic	380	400	341 or 191 and 193
N-Methyldecahydroquinolines	5	Bicyclic	233	283	Variable
Epiquinamide	1	Bicyclic	196	196	137
Histrionicotoxins	16	Bicyclic	291	235	96 or 250
Homopumiliotoxins	18	Bicyclic	223	353	180 and 84 or 207
Desmethylhomopumiliotoxins	4	Bicyclic	229	339	166 and 84
Deoxyhomopumiliotoxins	3	Bicyclic	193	251	164 and 84
Lehmizidines	9	Bicyclic	275	293	Variable
Other izidines	68	Bicyclic	191	295	Variable
Pumiliotoxins	39	Bicyclic	209	353	166 or 70 or 193
Allopumiliotoxins	23	Bicyclic	225	357	70 or 209
Deoxipumiliotoxins	15	Bicyclic	193	309	150
Dehydrodesmethylpumiliotoxins	4	Bicyclic	221	251	162 and 160
Desmethylpumiliotoxins	3	Bicyclic	249	265	152 and 170
Batrachotoxins	6	Steroidal	399	568	399
Indolic alkaloids	2	Indolic	346	346	346 and 173
Pseudophrynamines	16	Indolic	242	528	Variable
Piperidines	29	Monocyclic	183	269	98 or 114
Pyrrolidines	10	Monocyclic	183	279	Variable
Epibatidines	4	Pyridinic	208/210	308/310	69 or 82 and 56 and 167
Pyridinic alkaloids	3	Pyridinic	162	239	84
Cyclopentaquinazolines	9	Tricyclic	235	279	211
Gephyrotoxins	2	Tricyclic	287	289	Variable
Spiropyrrolizidines	7	Tricyclic	151	254	112 or 126 or 142
Tricyclics	66	Tricyclic	191	333	Variable
Alkaloids without classification	192	Variable	151	434	58 or 67 or 70 or 82 or 84 or 86 or 110 or 116 or 118 or 120 or 122
Total number of alkaloids	**851**				

*The number reported for each class/subclass is until of 2012

#### Data Derived from LC–MS

LC–MS have been recently employed for the separation and annotation of lipophilic alkaloids from Dendrobatoidea superfamily with low molecular weights. In contrast, this chromatographic method is the only possible option for the analysis of lipophilic alkaloids with high molecular weight, such as batrachotoxins, or hydrophilic alkaloids, such as TTXs.

For the lack of a LC–MS/MS based database of lipophilic alkaloids ionized by electrospray (ESI), the current strategy involved a comparison with the database created for a different platform. The annotation process from MS/MS spectra obtained by ESI should be informed by the Daly database designed for GC–MS characterized by electronic ionization (EI) (Daly et al. 2005) or from the commercial Dictionary of Natural Products (DNP v.27.2, http://dnp.chemnetbase.com). In High Resolution instruments, one possible strategy involves the creation of a personal library with the accurate masses of all frog alkaloids obtained from their molecular formulas. With this, is possible to track the corresponding product ions [M + H]^+^ on different chromatographic LC–MS/MS analyses of samples (McGugan et al. 2016; Protti-Sánchez et al. 2019; Fischer et al. 2020). As LC–MS is more sensitive than GC–MS, it offers the possibility of detecting a higher number of alkaloids. A correlation between the results using both platforms could improve the annotation rate. Results for LC–MS/MS could be informed from GC–MS fragmentation patterns when a molecular formula that belongs to several alkaloids is detected. GC–MS results could help to narrow-down the number of candidate structures. Similarly, some alkaloid detected by GC–MS and that belong to the same alkaloid family but have different molecular masses, could show similar (but never equivalent) fragmentation patterns, and LC–MS/MS analyses could support the annotation with the information of molecular ions. For low resolution LC–MS/MS analysis, the accurate mass and deconvolution of molecular formula is not feasible, and the remaining strategy is the comparison of the mass spectra obtained by ESI or DESI, with the fragments from the GC–MS database. Then, another useful tool, involves the generation of in silico MS/MS spectra for suspected compounds, using SMILES input from each structure in the in silico fragmentation tool CFM-ID v3.0 (available at https://cfmid3.wishartlab.com/) or CFM-ID v4.0 (https://cfmid.wishartlab.com/).

For the case of hydrophilic alkaloids, HPLC–MS allowed the determination of new TTX analogues by reporting new fragments (Pires et al. 2005; Rodríguez et al. 2012, 2017). Some tetrodotoxins and other amphibian hydrophilic alkaloids, their molecular formulas and precursor ions [M + H]^+^ are summarized in Table 5. Targeted analysis could be applied for the selective analysis of these compounds and annotations of level 1 could be obtained at least for ﻿TTX, ﻿4-epi TTX, 4,9-anhydro TTX and 5,6,11-deoxyTTX comparing their retention time and fragmentation patterns with analytical standards (Bane et al. 2014). However, there are more than 20 TTX analogues detected in natural sources, and in a similar way as occurs for lipophilic dendrobatoid alkaloids, the shortage of commercial standards is one of the major problems faced by researchers who study these chemicals.

**Table 5:** Molecular formula of some tetrodotoxins, zetekitoxins and chiriquitoxins, and their [M + H]^+^ precursor ions in mass spectrometry. Data obtained from (Yotsu et al. 1990; Yotsu-Yamashita et al. 2004; Otero et al. 2013)

Alkaloid	Molecular formula	[M + H]^+^ (m/z)
TTX	C_11_H_17_N_3_O_8_	320.1088
4-epiTTX	C_11_H_17_N_3_O_8_	320.1088
6-epiTTX	C_11_H_17_N_3_O_8_	320.1088
Tetrodonic acid	C_11_H_17_N_3_O_8_	320.1088
11-oxoTTX	C_11_H_17_N_3_O_9_	336.1038
4,9-anhydroTTX	C_11_H_15_N_3_O_7_	302.0983
6-epi-4,9-anhydroTTX	C_11_H_15_N_3_O_7_	302.0983
11-deoxyTTX	C_11_H_17_N_3_O_7_	304.1139
5-deoxyTTX	C_11_H_17_N_3_O_7_	304.1139
11-norTTX-6(S)-ol	C_10_H_15_N_3_O_7_	290.0983
11-norTTX-6(R)-ol	C_10_H_15_N_3_O_7_	290.0983
5,6,11-trideoxyTTX	C_10_H_13_N_3_O_6_	272.0877
4-epi-5,6,11-trideoxyTTX	C_10_H_13_N_3_O_6_	272.0877
6,11-dideoxyTTX	C_10_H_13_N_3_O_7_	288.0826
4,9-anhydro-5,6,11-trideoxyTTX	C_10_H_11_N_3_O_5_	254.0771
Zetekitoxin AB	C_16_H_24_N_8_O_12_S	553.1313
Chiriquitoxin	C_13_H_20_N_4_O_10_	393.1252

#### Recommendations for Annotation of Molecular Features

With the aim of improving ﻿transparency in measurement and metabolite annotation and documentation in the future we propose that in the annotation tables metabolite identifiers and unknown molecular features that could not be completely annotated. Including some chemical identifiers as CAS number, InChIKeys, theoretical retention indexes (RI) and IUPAC names in annotation tables would facilitate global interpretation. Molecular features that could not be annotated for the lack of a match with a reference library could be reported as unknown or unknown compounds from a specific chemical class if the annotation is level 3. To avoid subjectivity defining chemical classes we suggest ClassyFire chemical taxonomy (Feunang et al. 2016). This program uses only chemical structures and structural features to automatically assign all known chemical compounds to a taxonomy consisting of > 4800 different categories defined by unambiguous, computable structural rules. Each compound is classified in different levels such as Kingdom, SuperClass, Class, SubClass, etc. (Feunang et al. 2016). To perform batch ClassyFire classification there is a classification tool available from the Fienh Lab from the University of California Davis (https://cfb.fiehnlab.ucdavis.edu/) that only requires the InChIKey of each molecular feature as input.

There are other sources available that may facilitate annotation of compounds analyzed in dendrobatoids for both GC–MS and HPLC–MS. GNPS environment is one of the most versatile tools that allows to perform library search for data derived from GC–MS (https://ccms-ucsd.github.io/GNPSDocumentation/gcanalysis/), library search for data derived from LC–MS analysis (https://ccms-ucsd.github.io/GNPSDocumentation/featurebasedmolecularnetworking/), query a single MS/MS spectrum across all public GNPS datasets (https://ccms-ucsd.github.io/GNPSDocumentation/masst/), creation/publication of Spectral Libraries (https://ccms-ucsd.github.io/GNPSDocumentation/batchupload/) and more advanced tools (https://gnps.ucsd.edu/ProteoSAFe/static/gnps-splash.jsp?redirect=auth). Other reference spectra could be consulted in the following resources: the Dictionary of Natural Products (DNP) (https://dnp.chemnetbase.com/faces/chemical/ChemicalSearch.xhtml;jsessionid=871BB433A50A6C0FC01B9243717C9315), Pherobase (https://www.pherobase.com/), Human Metabolome Database (HMDB) (https://hmdb.ca/), METLIN (https://metlin.scripps.edu/landing_page.php?pgcontent=mainPage), MassBank Japan (http://www.massbank.jp/), MassBank Europe (https://massbank.eu/MassBank/), MassBank North America (https://mona.fiehnlab.ucdavis.edu/) Supernatural II (https://bioinf-applied.charite.de/supernatural_new/index.php), ChEMBL (https://www.ebi.ac.uk/chembl/), Mass Spectral and GC Data of Drugs, Poisons, Pesticides, vocBinBase (https://bitbucket.org/fiehnlab/binbase/src/master/), and ﻿mVOC database 3.0 (https://bioinformatics.charite.de/mvoc/) (Carazzone et al. 2021).

### Statistical Analysis

The Eco-metabolomics approach should involve the selection/measurement of different metavariables (i.e. ﻿other variables different than metabolites). Depending on the study some of them could be ecological (species, location, collection time, age, sex, confounding variables), or chemical (extraction method, type of metabolites, type of solvent, extraction time).

**Fig. 7:**
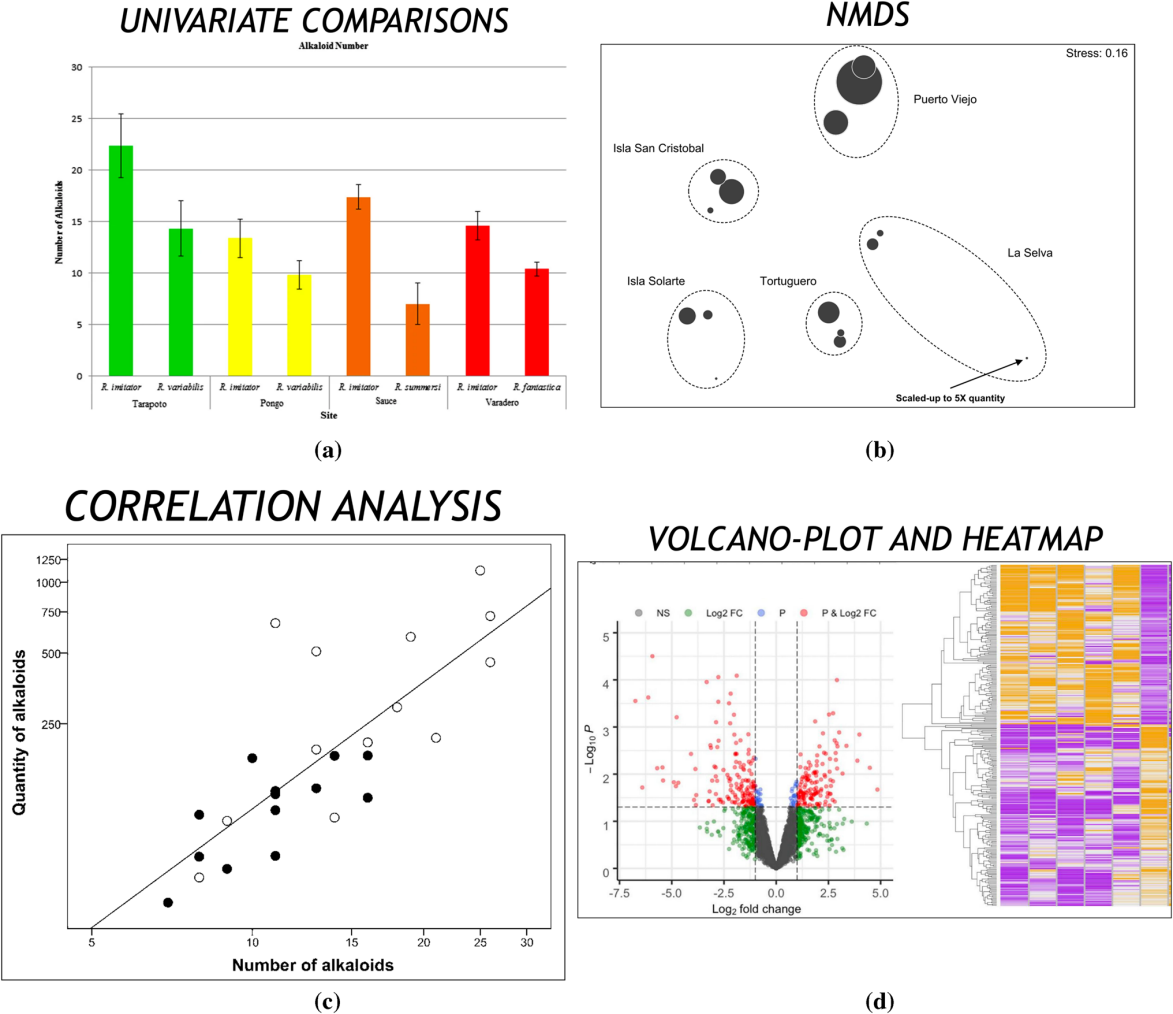
Univariate and multivariate Eco-metabolomics data analysis employed for studying chemical ecology from dendrobatoids. **A**. Univariate comparison between the number of alkaloids found in *Ranitomeya* species. Copyright © 2014, (Stuckert et al. 2014). **B**. NMDS obtained from *Oophaga pumilio* in five locations. Copyright © 2015, (Mina et al. 2015). **C**. Correlation analysis obtained between the number and the quantity of alkaloids from *Oophaga pumilio*. Reprinted (adapted) with permission from Saporito et al. 2010. Sex-related differences in alkaloid chemical defenses of the dendrobatoid frog *Oophaga pumilio* from Cayo Nancy, Bocas del Toro, Panama. J Nat Prod 73:317–321. doi:10.1021/np900702d . Copyright © 2010 American Chemical Society. Saporito et al. (Saporito et al. 2010). **D**. Volcano-plot and heatmap obtained from the proteomic analysis of intestine tissues of *Oophaga sylvatica*. Copyright © 2021, (O’Connell et al. 2021)

For studying the extrinsic process acting upon the sequestration and accumulation of alkaloids, different researchers have employed univariate and multivariate statistical analysis. They have compared chemical profiles from dendrobatoids to study the significance of metavariables such as population, species, location, age, sex, diet, color, and impact of habitat fragmentation. For univariate analysis they have compared quantities or chromatographic peak intensities/areas and performed parametric or non-parametric tests for measuring the significance of this differentiation employing ANOVA and Kruskal–Wallis analysis, respectively (Mina et al. 2015; Moskowitz et al. 2020), depending on the data-normality. For multivariate analysis, the main type of analysis used has been Non-metric multidimensional scaling (NMDS) accompanied by ANOSIM or PERMANOVA analysis for testing the significance of different metavariables in differentiating alkaloid profiles (Saporito et al. 2006, 2007, 2010; Stuckert et al. 2014; Mina et al. 2015; Moskowitz et al. 2022). Correlation analysis have also been tested for measuring the strength of the correlation between total amount of alkaloids and diversity of alkaloids (Saporito et al. 2010), a common pattern found on different species. Figure 7 illustrates some examples from these analyses. Other very powerful visualization tools for illustrating metabolomics patterns such as heatmaps, and volcano plots have been recently employed (Caty et al. 2019; Moskowitz et al. 2020; O’Connell et al. 2021).

Other less used statistical analysis has been hardly ever used in the Eco-metabolomics of poison frogs. Some of these analyses include fold change, t-test, significance analysis of metabolites (SAM), empirical Bayesian analysis of metabolites (EBAM), principal component analysis (PCA), partial least squares-discriminant analysis (PLS-DA), orthogonal partial least squares-discriminant analysis (OPLS-DA), K-means, self organizing map (SOM), random forests, support vector machine (SVM) and molecular networking analysis. As datasets usually include large number of features, the significance level of univariate analyses should be appropriately determined to reduce the number of false positives and false negatives. For reducing false positive, in univariate analysis familywise error rate (FWER) correction, such as a Bonferroni correction, is a conservative approach, in which the p- values are multiplied by the number of comparisons. In contrast, for reducing false negatives, false discovery rate (FDR) correction is a highly sensitive method (Walker 2013; Putri and Fukusaki 2014). For multivariate analysis there are non-supervised approaches and supervised approaches, which differ in how samples are grouped within the multivariate calculations. Non-supervised solely have access to the matrix of measurement before grouping samples. In contrast, supervised methods have access to qualitative or quantitative traits (e.g., species, location, body size, tissue type) and the matrix of measurements, for grouping samples. Molecular networking organizes metabolite features from a Metabolomics analysis into a connectivity network based on similarities in molecular fragmentation patterns obtained from mass spectrometry (Covington et al. 2017). This analysis clusters families of molecules through vector correlations between fragment ions and enhances the interpretation of metabolome differentiation using a chemically informed visualization. Also, it enhances the annotation process with experimental and in silico databases (Watrous et al. 2012). Molecular networking could be performed for both GC–MS (Wang et al. 2016; Aksenov et al. 2021) and LC–MS/MS (Wang et al. 2016; Nothias et al. 2020) data in the GNPS ecosystem. In the future, combining Metabolomics and Genomic analysis, molecular networking will probe to be useful to prioritize features. For example linking natural products from frogs with their cognate gene clusters/gene cluster families (Covington et al. 2017) associated with metabolite resistance, metabolism, sequestration or synthesis.

R studio (http://www.rstudio.org/) offer the possibility to perform statistical analysis in combination with packages as HybridMTest (http://bioconductor.org/packages/HybridMTest/) (Pounds and Fofana 2020), vegan (https://CRAN.R-project.org/package=vegan) (Jari et al. 2020), Rcmdr (https://cran.r-project.org/web/packages/Rcmdr/index.html) (Fox and Bouchet-Valat 2020), ggplot2 (https://cran.r-project.org/web/packages/ggplot2/index.html) (Wickham 2016), among others. Other platforms that facilitate visualization tools and exploratory statistical analysis are included in MZmine (http://mzmine.github.io/) (Pluskal et al. 2010), GNPS Dashboard (https://gnps-explorer.ucsd.edu/MSV000086521?dataset_accession=MSV000086521&metadata_source=DEFAULT&metadata_option =) (Petras et al. 2022), QIIME 2 (https://qiime2.org/) (Bolyen et al. 2019) and Metaboanalyst 5.0 (https://www.metaboanalyst.ca/) (Pang et al. 2022).

### Additional Analytical Challenges and Possible Solutions

#### Quantification and Semi-Quantification

Most of the current studies have been focused on a general profiling of metabolites rather than quantification. Semi-quantification of lipophilic alkaloids have been performed employing ﻿nicotine ((-)-nicotine 99%, ﻿Sigma-Aldrich, Milwaukee, WI) (Saporito et al. 2010; Garraffo et al. 2012), or D3-nicotine ﻿(Sigma- Aldrich, St. Louis, MO) (McGugan et al. 2016) as internal standards. The trans-decahydroquinoline 97% ﻿(Sigma- Aldrich, St. Louis, MO) could also be potentially used. Simultaneous quantitative analysis of batrachotoxin and epibatidine in plasma has been made possible employing LC–MS by the existence of commercial standards of these two substances (Shiraishi et al. 2017) and could be applied for quantification in frogs in the future. In contrast, for the quantification of hydrophilic alkaloids such as TTX analogues, TTX have been used as internal standard (Chen et al. 2011). For quantification of TTX, calibration curves with TTX or semi-quantification employing voglibose as internal standard have been employed (Kudo et al. 2012) (see Table 6).

**Table 6:** Chemical vendors of some specific compounds that could be used as analytical standards for the analysis of the chemical profiles of dendrobatoids

Alkaloid	Molecular weight	CAS number	Country of supplier	Supplier link	Quantity available
Tetrodotoxin	319.27	4368–28-9	USA	http://www.bocsci.com/tetrodotoxin-cas-4368-28-9-item-5-465663.html	Unspecified
Batrachotoxin	538.75	23,509–16-2	France	https://www.latoxan.com/moleculars_product.php?id=1266&n=1	10 ug -1 mg
trans-Decahydroquinoline	139.24	767–92-0	USA	https://www.sigmaaldrich.com/US/en/product/aldrich/766674	5 g
Epibatidine	281.61		USA	https://www.sigmaaldrich.com/US/en/product/sigma/e1145	5 g
Voglibose	267.28	83,480–29-9	USA	https://www.sigmaaldrich.com/US/en/product/sigma/50359	10 mg

Currently, two major problems make it cumbersome to make decisions regarding internal standards in dendrobatoid chemical ecology: (1) The lack of more commercial standards considering the great diversity of alkaloid structures found on frogs and (2) the lack of studies evaluating suppression effects of different analytical standards.

The low availability of analytical standards from most of dendrobatoid alkaloids is attributable to the unique natural sources where these compounds have been found: poison frogs (Daly et al. 2005), some birds (Dumbacher et al. 2000) and a few identified arthropods from where frogs sequester these alkaloids (Dumbacher et al. 2004; Saporito et al. 2006, 2012). In contrast to other groups of organisms, where a higher knowledge about the biosynthetic pathways or advances in their commercial synthesis had led to an easy availability, just a few chemical compounds from dendrobatoids could be commercially purchased. Table 6 summarizes one example of the chemical vendors available as analytical standards that could be used for the Eco-metabolomic analysis in poison frogs. Fortunately, through collaborations with organic chemist it has been possible to employed synthetic forms of PTX **251D** synthesized in research labs (Vandendriessche et al. 2008; Mebs et al. 2014) or companies (PepTech, Burlington, MA, USA) (Alvarez-Buylla et al. 2022), and the histrionichotoxin ﻿HTX **235A** (Jeckel et al. 2022).

Currently, there is a need for evaluating suppression effects of different analytical standards or isolated compounds obtained from fractionated extracts to determine its convenience in a determined experiment.

### Lack of Updated Databases for GC–MS and LC–MS Platforms

In a Metabolomic analysis is estimated that an average of only 2% of the data can be annotated (Aksenov et al. 2017). This is even a more common problem in metabolomics analysis of animals because most of the databases found in public repositories are specialized in human derived metabolites, and many reference databases exclude some molecular structures from animals. In consequence, analysis from non-model organisms, as do not have matches with previous entries of similar organisms on repositories or databases tend to have a higher number of truly novel compounds.

There is a need to construct open-source databases with the mass spectra of dendrobatoid metabolites, for both, GC–MS and LC–MS platforms. These databases will improve the annotation task of dendrobatoid metabolites, facilitating the automatic comparison with experimental data on both chromatographic systems. GC–MS database (Daly et al. 2005) needs to be updated, incorporating more diagnostic fragments and linear retention indices (RI). The creation of the first open LC–MS/MS database is essential in the coming years. This will speed up data analysis and probably motivate that more researchers use this system.

## Future Perspectives

Many mysteries remain regarding the ecology and chemistry of frogs from the superfamily Dendrobatoidea. Biological diversity keeps growing because new species are still being discovered. However, the number of identified compounds has reached a plateau. This could be partially explained because many new metabolites are being detected at trace levels and their isolation and further annotation is sometimes virtually impossible. It is expected that a combined effort between biologists and chemists will lead to an increment in the chemical diversity of this superfamily including annotated and non-annotated compounds in the upcoming analyses. Also, the large color variation among dendrobatoid phenotypes that have amazed biologists interested in understanding aposematism, has affected the way how science is performed. Currently, we have a better knowledge about conspicuous species than cryptically colored (brownish) ones, and we need to gain more knowledge about the latter for understanding the evolution of chemical defences in this group. In a similar way, chemical defences were reduced to a synonym of alkaloid. As dendrobatoid alkaloids had unique structures, are known to be actively sequestered and have promising therapeutical applications, most of the studies were focused on improving the available methods for extracting and detecting alkaloids. This resulted in a lack of motivation for extracting metabolites different from alkaloids and a reduction on the spectrum of compounds understood as chemical defences within the superfamily Dendrobatoidea. Studies with other amphibians have demonstrated that chemical defences could include proteins, peptides, volatile organic compounds (VOCs), and we need to incorporate extraction and characterization methods oriented to analyze metabolites others than alkaloids. Little efforts have compared extractions methods and platforms. To improve the recovery of most of the compounds, future investigations should be done studying the extraction efficiency of different methods, evaluating their sensitivity, specificity, and matrix interferences. Using an Eco-metabolomics approach, we will be able to understand why not all individuals are equally defended, studying the significance of ecological (species, location, time, sex, age, etc.), and chemical metavariables (extraction methods, type of metabolites, etc.). On the top of that, longitudinal analyses comparing the chemical profiles of individual specimens across time (day vs. night, or months of the year, or life-stages) in their natural habitats or subjected to manipulative experiments are not feasible right now. The lethal gold standard method restricts to chemical analyses to a single sampling moment. There is a need to develop a non-lethal method that allows a proper estimation of how variable was the chemical profile of a specimen in the past, or how it could change after certain manipulation in the future.

To achieve this goal, it is necessary to standardize a workflow and to insist about the importance of sharing raw data from the chromatographic analyses derived from species from this superfamily. Future analyses applying a consensus-based Eco-metabolomics workflow could enhance the extraction of biologically relevant information from the chemical profiles and their comparisons among species and studies. Sharing raw data in public repositories from chemical profiles already published, will enable to construct a single meta-analysis aimed to compare dendrobatoid chemodiversity, including annotated and non-annotated metabolites. To illustrate how scattered is this chemical information about dendrobatoids, Online Resource 1 summarizes the most meaningful chemical discoveries made for different alkaloids’ families. Online Resource 1 demonstrates how the lack of a consensus-based Eco-metabolomics workflow, and the unavailability of chromatograms and mass spectra has slowed down the progress of the chemical ecology. This invaluable chemical information is dispersed in several published papers and their supplementary material, and needs to be summarized. Establishing long-lasting collaborations with organic chemists will also increase the number of level 1 annotations, improving the availability of analytical standards of different chemical structures.

The application of an Eco-metabolomics approach to the chemical ecology to the superfamily Dendrobatoidea will be paramount for studying other biological mysteries that remain unsolved. Alkaloid sequestration mechanism remained as a black box for several years. Alkaloids get-in trough diet and are accumulated on frog skin, but how are alkaloids actively transported from the digestive system to the skin? Just recently, evidence about the altered expression of RNA and proteins involved in this process has been found (Caty et al. 2019; O’Connell et al. 2021). How frogs’ metabolism evolved to cope with these toxic compounds and resist a possible intoxication? Mutations in channels and receptors are giving some insights about it (Tarvin et al. 2016, 2017; Márquez et al. 2019), but do not govern completely the process of physiological auto-resistance (Abderemane-Ali et al. 2021; Márquez 2021). A multi-omics approach will be needed to fully comprehend the difference between detoxification and sequestration mechanism. How traits of aposematic system evolved with chemical defences and specific predator pressures? What is the toxic/noxious/unpalatable/repellent functions of different metabolites? What types of multimodal communication exist within chemically defended dendrobatoid species in conspecifics and heterospecific contexts? Just now, scientists are starting to study this question (Stynoski and Noble 2012; Amézquita et al. 2017; Rojas 2017; Rojas et al. 2018), and new metabolites will lead to new behavioral experiments aimed to understand their ecological functions. Future collaborations between biologists and chemists, researchers from different expertise and backgrounds will improve the exploration of these questions and many more regarding the chemical defences from the Dendrobatoidea superfamily.

### Supplementary Information

Below is the link to the electronic supplementary material.

Supplementary file1 (XLSX 510 KB)

## References

[CR1] Abderemane-Ali F, Rossen ND, Kobiela ME (2021). Evidence that toxin resistance in poison birds and frogs is not rooted in sodium channel mutations and may rely on “toxin sponge” proteins. J Gen Physiol.

[CR2] Aksenov AA, Da Silva R, Knight R (2017). Global chemical analysis of biology by mass spectrometry. Nat Rev Chem.

[CR3] Aksenov AA, Laponogov I, Zhang Z (2021). Auto-deconvolution and molecular networking of gas chromatography–mass spectrometry data. Nat Biotechnol.

[CR4] Albuquerque EX, Daly JW, Witkop B (1971). Batrachotoxin: chemistry and pharmacology. Science.

[CR5] Alseekh S, Aharoni A, Brotman Y (2021). Mass spectrometry-based metabolomics: a guide for annotation, quantification and best reporting practices. Nat Methods.

[CR6] Alvarez-Buylla A, Payne CY, Vidoudez C (2022). Molecular physiology of pumiliotoxin sequestration in a poison frog. PLoS One.

[CR7] Alvarez-Buylla A, Moya-Garzon MD, Rangel AE, et al (2023) Binding and sequestration of poison frog alkaloids by a plasma globulin. bioRxiv 2022.11.22.517437. 10.1101/2022.11.22.517437 10.7554/eLife.85096 PMC10783871 38206862

[CR8] Amézquita A, Ramos Ó, González MC (2017). Conspicuousness, color resemblance, and toxicity in geographically diverging mimicry: The pan-Amazonian frog *Allobates femoralis*. Evolution (n y).

[CR9] Asakawa M, Shida Y, Miyazawa K, Noguchi T, de Azevedo CL (2012). Instrumental analysis of tetrodotoxin. Chromatography - The most versatile method of chemical analysis.

[CR10] Bane V, Lehane M, Dikshit M (2014). Tetrodotoxin: Chemistry, toxicity, source, distribution and detection. Toxins (basel).

[CR11] Betancourth-Cundar M, Palacios-Rodríguez P, Mejía-Vargas D (2020). Genetic differentiation and overexploitation history of the critically endangered Lehmann’s poison frog: *Oophaga lehmanni*. Conserv Genet.

[CR12] Bolyen E, Rideout JR, Dillon MR (2019). Reproducible, interactive, scalable and extensible microbiome data science using QIIME 2. Nat Biotechnol.

[CR13] Bonat Celli G, Ghanem A, Su-Ling Brooks M (2016). Influence of freezing process and frozen storage on the quality of fruits and fruit products. Food Rev Int.

[CR14] Brunetti AE, Merib J, Carasek E (2015). Frog volatile compounds: Application of *in vivo* SPME for the characterization of the odorous secretions from two species of *Hypsiboas* treefrogs. J Chem Ecol.

[CR15] Carasek E, Bernardi G, Morelli D, Merib J (2021). Sustainable green solvents for microextraction techniques: Recent developments and applications. J Chromatogr A.

[CR16] Carazzone C, PG Rodríguez J, Gonzalez M, López GD (2021) Volatilomics of natural products: Whispers from nature. In: Zhan (ed) Metabolomics: Methodology and Applications in Medical Sciences and Life Sciences. Intech Open 2–25. 10.5772/intechopen.97228

[CR17] Cardall BL, Brodie ED, Brodie ED, Hanifin CT (2004). Secretion and regeneration of tetrodotoxin in the rough-skin newt (*Taricha granulosa*). Toxicon.

[CR18] Carvajal-Castro JD, Vargas-Salinas F, Casas-Cardona S (2021). Aposematism facilitates the diversification of parental care strategies in poison frogs. Sci Rep.

[CR19] Caty SN, Alvarez-Buylla A, Byrd GD (2019). Molecular physiology of chemical defenses in a poison frog. J Exp Biol.

[CR20] Chen XW, Liu HX, Jin YB (2011). Separation, identification and quantification of tetrodotoxin and its analogs by LC-MS without calibration of individual analogs. Toxicon.

[CR21] Cipriani I, Rivera M (2009). Detección de alcaloides en la piel de cuatro especies de anfibios ecuatorianos (Anura: Dendrobatidae). Rev Ecuat Med Cienc Biol.

[CR22] Clark VC, Rakotomalala V, Ramilijaona O (2006). Individual variation in alkaloid content of poison frogs of Madagascar (*Mantella*; Mantellidae). J Chem Ecol.

[CR23] Covington BC, McLean JA, Bachmann BO (2017). Comparative mass spectrometry-based metabolomics strategies for the investigation of microbial secondary metabolites. Nat Prod Rep.

[CR24] Crothers L, Saporito RA, Yeager J (2016). Warning signal properties covary with toxicity but not testosterone or aggregate carotenoids in a poison frog. Evol Ecol.

[CR25] Da Silva RR, Dorrestein PC, Quinn RA (2015). Illuminating the dark matter in metabolomics. Proc Natl Acad Sci U S A.

[CR26] Daly JW (1995). The chemistry of poisons in amphibian skin. Proc Natl Acad Sci U S A.

[CR27] Daly JW (2003). Ernest Guenther award in chemistry of natural products. Amphibian skin: A remarkable source of biologically active arthropod alkaloids. J Med Chem.

[CR28] Daly JW, Myers CW (1967). Toxicity of panamanian poison frogs (*Dendrobates*): Some biological and chemical aspects. Science.

[CR29] Daly J, Witkop B (1971). Batrachotoxin, an extremely active cardio- and neurotoxin from the Colombian arrow poison frog *Phyllobates aurotaenia*. Clin Toxicol.

[CR30] Daly JW, Witkop B, Bommer P, Biemann K (1965). Batrachotoxin. The active principle of the Colombian arrow poison frog. Phyllobates Bicolor J Am Chem Soc.

[CR31] Daly JW, Tokuyama T, Habermehl G (1969). Froschgifte. Isolierung und Struktur von Pumiliotoxin C. Justus Liebigs Ann Chem.

[CR32] Daly JW, Karle I, Myers CW (1971). Histrionicotoxins: Roentgen-Ray analysis of the novel allenic and acetylenic spiroalkaloids isolated from a Colombian frog, *Dendrobates histrionicus*. Proc Natl Acad Sci.

[CR33] Daly JW, Witkop B, Tokuyama T (1977). Gephyrotoxins, Histrionicotoxins and Pumiliotoxins from the Neotropical frog *Dendrobates histrionicus*. Helv Chim Acta.

[CR34] Daly JW, Brown GB, Mensah-Dwumah M, Myers CW (1978). Classification of skin alkaloids from neotropical poison-dart frogs (Dendrobatidae). Toxicon.

[CR35] Daly JW, Tokuyama T, Fujiwara T (1980). A new class of indolizidine alkaloids from the poison frog, *Dendrobates tricolor*. X-ray analysis of 8-hydroxy-8-methyl-6-(2’-methylhexylidene)-1-azabicyclo[4.3.0]nonane. J Am Chem Soc.

[CR36] Daly JW, Spande TF, Whittaker N (1986). Alkaloids from dendrobatid frogs: structures of two w-nydroxy congeners of 3-butyl-5-propylindolizidine and occurrence of 2,6-distributed pyrrolidines and a 2,6-distributed piperidine. J Nat Prod.

[CR37] Daly JW, Myers CW, Whittaker N (1987). Further classification of skin alkaloids from neotropical poison frogs (Dendrobatidae), with a general survey of toxic/noxious substances in the amphibia. Toxicon.

[CR38] Daly JW, Mcneal E, Gusovsky F (1988). Pumiliotoxin alkaloids: Relationship of cardiotonic activity to sodium channel activity and phosphatidylinositol turnover. J Med Chem.

[CR39] Daly J, Gusovsky F, Myers CW (1994). First occurrence of tetrodotoxin in a dendrobatid frog (*Colostethus inguinalis*), with further reports for the bufonid genus *Atelopus*. Toxicon.

[CR40] Daly J, Martin Garraffo H, Spande TF (1994). Dietary source for skin alkaloids of poison frogs (Dendrobatidae)?. J Chem Ecol.

[CR41] Daly J, Secunda SI, Garraffo HM (1994). An uptake system for dietary alkaloids in poison frogs (Dendrobatidae). Toxicon.

[CR42] Daly JW, Kaneko T, Wilham J (2002). Bioactive alkaloids of frog skin: Combinatorial bioprospecting reveals that pumiliotoxins have an arthropod source. Proc Natl Acad Sci U S A.

[CR43] Daly JW, Garraffo HM, Spande TF (2003). Evidence for an enantioselective pumiliotoxin 7-hydroxylase in dendrobatid poison frogs of the genus *Dendrobates*. Proc Natl Acad Sci U S A.

[CR44] Daly JW, Spande TF, Garraffo HM (2005). Alkaloids from Amphibian skin: A tabulation of over eight-hundred compounds. J Nat Prod.

[CR45] Daly JW, Ware N, Saporito RA (2009). N-Methyldecahydroquinolines: An unexpected class of alkaloids from Amazonian poison frogs (Dendrobatidae). J Nat Prod.

[CR46] Daly JW, Garraffo HM, Spande TF (1993) Amphibian Alkaloids. In: Cordell G (ed) The alkaloids. Chemistry and pharmacology. Academic Press, San Diego, pp 185–288. 10.1016/S0099-9598(08)60136-4

[CR47] Darst CR, Menéndez-Guerrero PA, Coloma LA, Cannatella DC (2005). Evolution of dietary specialization and chemical defense in poison frogs (Dendrobatidae): A comparative analysis. Am Nat.

[CR48] Darst CR, Cummings ME, Cannatella DC (2006). A mechanism for diversity in warning signals: Conspicuousness versus toxicity in poison frogs. Proc Natl Acad Sci.

[CR49] Dumbacher JP, Spande TF, Daly JW (2000). Batrachotoxin alkaloids from passerine birds: A second toxic bird genus (*Ifrita kowaldi*) from New Guinea. Proc Natl Acad Sci.

[CR50] Dumbacher JP, Wako A, Derrickson SR (2004). Melyrid beetles (*Choresine*): A putative source for the batrachotoxin alkaloids found in poison-dart frogs and toxic passerine birds. Proc Natl Acad Sci.

[CR51] Edwards MW, Daly JW, Myers CW (1988). Alkaloids from a panamanian poison frog, *Dendrobates speciosus*: Identification of pumiliotoxin-A and allo-pumiliotoxin class alkaloids, 3,5-disubstituted indolizidines, 5-substituted 8-methylindolizidines, and a 2-methyl-6-nonyl-4-hydroxypiperidine. J Nat Prod.

[CR52] Feunang YD, Eisner R, Knox C (2016). ClassyFire: Automated chemical classification with a comprehensive, computable taxonomy. J Cheminform.

[CR53] Fischer EK, Roland AB, Moskowitz NA (2019). The neural basis of tadpole transport in poison frogs. Proc R Soc B Biol Sci.

[CR54] Fischer EK, Alvarez H, Lagerstrom KM (2020). Neural correlates of winning and losing fights in poison frog tadpoles. Physiol Behav.

[CR55] Fitch RW, Garraffo HM, Spande TF (2003). Bioassay-guided isolation of epiquinamide, a novel quinolizidine alkaloid and nicotinic agonist from an Ecuadoran poison frog, *Epipedobates tricolor*. J Nat Prod.

[CR56] Fitch RW, Spande TF, Garraffo HM (2010). Phantasmidine: An epibatidine congener from the ecuadorian poison frog *Epipedobates anthonyi*. J Nat Prod.

[CR57] Fox J, Bouchet-Valat M (2020) Rcmdr: R Commander. In: R Packag. version 2.7–1. Package accessed in may 2021. https://cran.r-project.org/web/packages/Rcmdr/index.html

[CR58] Galeano SP, Harms KE (2016). Coloration in the polymorphic frog *Oophaga pumilio* associates with level of aggressiveness in intraspecific and interspecific behavioral interactions. Behav Ecol Sociobiol.

[CR59] Garraffo HM, Spande TF, Daly JW (1993). Alkaloids from bufonid toads (*Melanophryniscus*): Decahydroquinolines, pumiliotoxins and homopumiliotoxins, indolizidines, pyrrolizidines, and quinolizidines. J Nat Prod.

[CR60] Garraffo HM, Andriamaharavo NR, Vaira M (2012). Alkaloids from single skins of the Argentinian toad *Melanophryniscus rubriventris* (Anura, Bufonidae): An unexpected variability in alkaloid profiles and a profusion of new structures. Springerplus.

[CR61] Gonzalez M, Palacios-rodriguez P, Hernandez-restrepo J, et al (2021) First characterization of toxic alkaloids and volatile organic compounds ( VOCs ) in the cryptic dendrobatid *Silverstoneia punctiventris*. Front Zool 18(39):1–15. 10.1186/s12983-021-00420-1 10.1186/s12983-021-00420-1 PMC8390233 34446035

[CR62] Grant T (2004). On the Identities of *Colostethus inguinalis* (Cope, 1868) and *C. panamensis* (Dunn, 1933), with Comments on *C. latinasus* (Cope, 1863) (Anura: Dendrobatidae). Am Museum Novit.

[CR63] Grant T, Frost DR, Caldwell JP (2006). Phylogenetic systematics of dart-poison frogs and their relatives (Amphibia: Athesphatanura: Dendrobatidae). Bull Am Museum Nat Hist.

[CR64] Grant T, Rada M, Anganoy-Criollo M (2017). Phylogenetic systematics of dart-poison frogs and their relatives revisited (Anura: Dendrobatoidea). South Am J Herpetol.

[CR65] Grant,  (2007). A new, toxic species of *Colostethus* from the Cordillera Central of Colombia. Zootaxa.

[CR66] Guillory WX, Muell MR, Summers K, Brown JL (2019). Phylogenomic reconstruction of the Neotropical poison frogs (Dendrobatidae) and their conservation. Diversity.

[CR67] Hanifin CT (2010). The chemical and evolutionary ecology of tetrodotoxin (TTX). Toxicity in terrestrial vertebrates. Mar Drugs.

[CR68] Hantak MM, Grant T, Reinsch S (2013). Dietary alkaloid sequestration in a poison frog: An experimental test of alkaloid uptake in *Melanophryniscus stelzneri* (Bufonidae). J Chem Ecol.

[CR69] Haug K, Salek RM, Conesa P (2013). MetaboLights - An open-access general-purpose repository for metabolomics studies and associated meta-data. Nucleic Acids Res.

[CR70] Honerjäger P, Reiter M (1977). The cardiotoxic effect of batrachotoxin. Naunyn Schmiedebergs Arch Pharmacol.

[CR71] Horie M, Kobayashi S, Shimizu N, Nakazawa H (2002). Determination of tetrodotoxin in puffer-fish by Liquid Chromatography-Electrospray Ionization Mass Spectrometry. Analyst.

[CR72] Ibáñez R, Smith EM (1995). Systematic Status of Colostethus flotator and C. nubicola (Anura : Dendrobatidae) in Panama. Copeia.

[CR73] Jari O, Blanchet FG, Friendly M, et al (2020) R, vegan. In: Community Ecol. Package. Package accessed in may 2021. https://cran.r-project.org/package=vegan

[CR74] Jarmusch AK, Wang M, Aceves CM (2020). ReDU: a framework to find and reanalyze public mass spectrometry data. Nat Methods.

[CR75] Jeckel AM, Grant T, Saporito RA (2015). Sequestered and synthesized chemical defenses in the poison frog *Melanophryniscus moreirae*. J Chem Ecol.

[CR76] Jeckel AM, Kocheff S, Saporito RA, Grant T (2019). Geographically separated orange and blue populations of the Amazonian poison frog *Adelphobates galactonotus* (Anura, Dendrobatidae) do not differ in alkaloid composition or palatability. Chemoecology.

[CR77] Jeckel AM, Matsumura K, Nishikawa K (2020). Use of whole-body cryosectioning and desorption electrospray ionization mass spectrometry imaging to visualize alkaloid distribution in poison frogs. J Mass Spectrom.

[CR78] Jeckel AM, Bolton SK, Waters KR (2022). Dose-dependent alkaloid sequestration and N-methylation of decahydroquinoline in poison frogs. J Exp Zool Part A Ecol Integr Physiol.

[CR79] Jiang Y, Xi X, Ge L (2014). Bradykinin-related peptides (BRPs) from skin secretions of three genera of phyllomedusine leaf frogs and their comparative pharmacological effects on mammalian smooth muscles. Peptides.

[CR80] Jones T, Gorman J (1999). Further alkaloids common to ants and frogs: Decahydroquinolines and a quinolizidine. J Chem Ecol.

[CR81] Jones TH, Adams RMM, Spande TF (2012). Histrionicotoxin alkaloids finally detected in an ant. J Nat Prod.

[CR82] Kayaalp SO, Albuquerque EX, Warnick JE (1970). Ganglionic and cardiac actions of batrachotoxin. Eur J Pharmacol.

[CR83] Krieger AC, Povilaitis SC, Gowda P (2022). Noninvasive detection of chemical defenses in poison frogs using the MasSpec Pen. ACS Meas Sci Au.

[CR84] Kudo Y, Yasumoto T, Konoki K (2012). Isolation and structural determination of the first 8-epi-type tetrodotoxin analogs from the newt, *Cynops ensicauda popei*, and comparison of tetrodotoxin analogs profiles of this newt and the puffer fish, *Fugu poecilonotus*. Mar Drugs.

[CR85] Lam W, Ramanathan R (2002). In electrospray ionization source hydrogen/deuterium exchange LC-MS and LC-MS/MS for characterization of metabolites. J Am Soc Mass Spectrom.

[CR86] Liu X, Locasale JW (2017). Metabolomics: A Primer. Trends Biochem Sci.

[CR87] Mahmud Y, Yamamori K, Noguchi T (1999). Toxicity and tetrodotoxin as the toxic principle of a brackish water puffer, *Tetraodon steindachneri*, collected from Thailand. Food Hyg Saf Sci (Shokuhin Eiseigaku Zasshi).

[CR88] Man CN, Noor NM, Harn GL (2010). Screening of tetrodotoxin in puffers using gas chromatography-mass spectrometry. J Chromatogr A.

[CR89] Märki F, Witkop B (1963). The venom of the Colombian arrow poison frog *Phyllobates bicolor*. Experientia.

[CR90] Márquez R (2021). How do batrachotoxin-bearing frogs and birds avoid self intoxication?. J Gen Physiol.

[CR91] Márquez R, Ramírez-Castañeda V, Amézquita A (2019). Does batrachotoxin autoresistance coevolve with toxicity in *Phyllobates* poison-dart frogs?. Evolution (n y).

[CR92] Martin HC, Ibáñez R, Nothias LF (2020). Metabolites from microbes isolated from the skin of the panamanian rocket frog *Colostethus panamansis* (Anura: Dendrobatidae). Metabolites.

[CR93] McGugan JR, Byrd GD, Roland AB (2016). Ant and mite diversity drives toxin variation in the little devil poison frog. J Chem Ecol.

[CR94] Mebs D (2001). Toxicity in animals. Trends in evolution?. Toxicon.

[CR95] Mebs D, Pogoda W (2005). Variability of alkaloids in the skin secretion of the European fire salamander (*Salamandra salamadra terrestris*). Toxicon.

[CR96] Mebs D, Alvarez JV, Pogoda W (2014). Poor alkaloid sequestration by arrow poison frogs of the genus *Phyllobates* from Costa Rica. Toxicon.

[CR97] Mebs D, Yotsu-Yamashita M, Pogoda W (2018). Lack of alkaloids and tetrodotoxin in the neotropical frogs *Allobates* spp. (Aromobatidae) and *Silverstoneia flotator* (Dendrobatidae). Toxicon.

[CR98] Mina AE, Ponti AK, Woodcraft NL (2015). Variation in alkaloid-based microbial defenses of the dendrobatid poison frog *Oophaga pumilio*. Chemoecology.

[CR99] Mortari MR, Schwartz ENF, Schwartz CA (2004). Main alkaloids from the Brazilian dendrobatidae frog *Epipedobates flavopictus*: Pumiliotoxin 251D, histrionicotoxin and decahydroquinolines. Toxicon.

[CR100] Mosher HS, Fuhrman FA, Buchwald HD, Fischer HG (1964). Tarichatoxin-Tetrodotoxin: A Potent Neurotoxin. Science (80- ).

[CR101] Moskowitz NA, Dorritie B, Fay T (2020). Land use impacts poison frog chemical defenses through changes in leaf litter ant communities. Neotrop Biodivers.

[CR102] Moskowitz NA, Agui RD, Connell LAO (2022). Poison frog dietary preference depends on prey type and alkaloid load. Plos one.

[CR103] Mushtaq MY, Choi YH, Verpoorte R, Wilson EG (2014). Extraction for metabolomics: Access to the metabolome. Phytochem Anal.

[CR104] Myers CW, Daly J (1976). Preliminary evaluation of skin toxins and vocalizations in taxonomic and evolutionary studies of poison-dart frogs (Dendrobatidae). Bull Am Museum Nat Hist.

[CR105] Myers CW, Daly JW (1976). A new species of poison frog (Dendrobates) from Andean Ecuador, including an analysis of its skin toxins. Occas Pap Museum Nat Hist Univ Kansas, Lawrence, Kansas.

[CR106] Myers CW, Daly JW, Malkin B (1978). A dangerously toxic new frog (*Phyllobates*) used by Emberá indians of Western Colombia, with discussion of blowgun fabrication and dart poisoning. Bull Am Museum Nat Hist.

[CR107] Myers CW, Paolillo A, Daly JW (1991). Discovery of a defensively malodorous and nocturnal frog in the family Dendrobatidae : Phylogenetic significance of a new genus and species from the Venezuelan Andes. Am Museum Novit.

[CR108] Myers CW, Daly JW, Garraffo HM (1995). Discovery of the Costa Rican poison frog *Dendrobates granuliferus* in sympatry with *Dendrobates pumilio*, and comments on taxonomic use of skin alkaloids. Am Museum Novit.

[CR109] Nothias LF, Petras D, Schmid R (2020). Feature-based molecular networking in the GNPS analysis environment. Nat Methods.

[CR110] O’Connell LA, Course LISL, O’Connell JD (2021). Rapid toxin sequestration modifies poison frog physiology. J Exp Biol.

[CR111] Otero P, Rodríguez P, Botana AM, Fanali S, Haddad PR, Poole CF (2013). Analysis of natural toxins. Liquid Chromatography: Applications.

[CR112] Pang Z, Chong J, Zhou G (2021). MetaboAnalyst 5.0: Narrowing the gap between raw spectra and functional insights. Nucleic Acids Res.

[CR113] Pang Z, Zhou G, Ewald J (2022). Using MetaboAnalyst 5.0 for LC–HRMS spectra processing, multi-omics integration and covariate adjustment of global metabolomics data. Nat Protoc.

[CR114] Peters K, Worrich A, Weinhold A (2018). Current challenges in plant Eco-Metabolomics. Int J Mol Sci.

[CR115] Petras D, Phelan VV, Acharya D (2022). GNPS Dashboard: collaborative exploration of mass spectrometry data in the web browser. Nat Methods.

[CR116] Pires OR, Sebben A, Schwartz EF (2005). Further report of the occurrence of tetrodotoxin and new analogues in the Anuran family Brachycephalidae. Toxicon.

[CR117] Pluskal T, Castillo S, Villar-Briones A, Orešič M (2010). MZmine 2: Modular framework for processing, visualizing, and analyzing mass spectrometry-based molecular profile data. BMC Bioinformatics.

[CR118] Pounds S, Fofana D (2020) HybridMTest: Hybrid Multiple Testing. In: R Packag. version 1.34.0. Package accessed in may 2021. https://bioconductor.org/packages/HybridMTest/

[CR119] Protti-Sánchez F, Quirós-Guerrero L, Vásquez V (2019). Toxicity and alkaloid profiling of the skin of the Golfo Dulcean poison frog *Phyllobates vittatus* (Dendrobatidae). J Chem Ecol.

[CR120] Putri SP, Fukusaki E (2014). Mass Spectrometry-based Metabolomics.

[CR121] Raguso R, Agrawal A, Douglas A (2015). The raison d ’ e ˆ tre of chemical ecology. Ecology.

[CR122] Roberts LD, Souza AL, Gerszten RE, Clish CB (2012). Targeted metabolomics. Curr Protoc Mol Biol.

[CR123] Rodríguez P, Alfonso A, Otero P (2012). Liquid chromatography-mass spectrometry method to detect tetrodotoxin and its analogues in the puffer fish *Lagocephalus sceleratus* (Gmelin, 1789) from European waters. Food Chem.

[CR124] Rodríguez I, Alfonso A, Alonso E (2017). The association of bacterial C9-based TTX-like compounds with *Prorocentrum minimum* opens new uncertainties about shellfish seafood safety. Sci Rep.

[CR125] Rojas B (2017). Behavioural, ecological, and evolutionary aspects of diversity in frog colour patterns. Biol Rev.

[CR126] Rojas B, Rautiala P, Mappes J (2014). Differential detectability of polymorphic warning signals under varying light environments. Behav Processes.

[CR127] Rojas B, Burdfield-Steel E, De Pasqual C (2018). Multimodal aposematic signals and their emerging role in mate attraction. Front Ecol Evol.

[CR128] Samgina TY, Tolpina MI, Hakalehto E (2016). Proteolytic degradation and deactivation of amphibian skin peptides obtained by electrical stimulation of their dorsal glands. Anal Bioanal Chem.

[CR129] Samgina TY, Kovalev SV, Tolpina MD (2018). EThcD Discrimination of isomeric leucine/isoleucine residues in sequencing of the intact skin frog peptides with intramolecular disulfide bond. J Am Soc Mass Spectrom.

[CR130] Santos JC, Cannatella DC (2011). Phenotypic integration emerges from aposematism and scale in poison frogs. Proc Natl Acad Sci.

[CR131] Santos JC, Baquero M, Barrio-Amorós C (2014). Aposematism increases acoustic diversification and speciation in poison frogs. Proc R Soc B Biol Sci.

[CR132] Santos JC, Tarvin RD, O’Connell LA, Schulte BA, Goodwin TE, Ferkin MH (2016). A review of chemical defense in poison frogs (Dendrobatidae): Ecology, pharmacokinetics, and autoresistance. Chemical signals in vertebrates 13.

[CR133] Saporito RA, Donnelly MA, Garraffo HM (2006). Geographic and seasonal variation in alkaloid-based chemical defenses of *Dendrobates pumilio* from Bocas del Toro, Panama. J Chem Ecol.

[CR134] Saporito RA, Donnelly MA, Jain P (2007). Spatial and temporal patterns of alkaloid variation in the poison frog *Oophaga pumilio* in Costa Rica and Panama over 30 years. Toxicon.

[CR135] Saporito RA, Spande TF, Garraffo HM, Donnelly MA (2009). Arthropod alkaloids in poison frogs: A review of the “dietary hypothesis”. Heterocycles.

[CR136] Saporito RA, Donnelly MA, Madden AA (2010). Sex-related differences in alkaloid chemical defenses of the dendrobatid frog *Oophaga pumilio* from Cayo Nancy, Bocas del Toro, Panama. J Nat Prod.

[CR137] Saporito RA, Donnelly MA, Spande TF, Garraffo HM (2012). A review of chemical ecology in poison frogs. Chemoecology.

[CR138] Saporito RA, Grant T (2018) Comment on Amézquita et al. (2017) “Conspicuousness, color resemblance, and toxicity in geographically diverging mimicry: The pan-Amazonian frog *Allobates femoralis*.” Evolution (N Y) 72:1009–1014. 10.1111/evo.13468 10.1111/evo.13468 29524217

[CR139] Scheele BC, Pasmans F, Skerratt LF (2019). Amphibian fungal panzootic causes catastrophic and ongoing loss of biodiversity. Science (80- ).

[CR140] Schulte LM, Saporito RA, Davison I, Summers K (2017). The palatability of Neotropical poison frogs in predator-prey systems: Do alkaloids make the difference?. Biotropica.

[CR141] Sciarrone D, Pantò S, Ragonese C (2015). Evolution and status of preparative gas chromatography as a green sample-preparation technique. TrAC - Trends Anal Chem.

[CR142] Scott Chilton W, Bigwood J, Jensen RE (1979). Psilocin, bufotenine and serotonin: Historical and biosynthetic observations. J Psychoactive Drugs.

[CR143] Shiraishi Y, Ogawa T, Suzuki T (2017). Simultaneous quantification of batrachotoxin and epibatidine in plasma by Ultra-performance liquid chromatography / tandem mass spectrometry. Leg Med.

[CR144] Sillen-Tullberg B, Bryant S-T (1983). The evolution of aposematic coloration in distasteful prey: An individual selection model. Evolution (n y).

[CR145] Smirnov A, Qiu Y, Jia W (2019). ADAP-GC 4.0: Application of Clustering-Assisted Multivariate Curve Resolution to Spectral Deconvolution of Gas Chromatography-Mass Spectrometry Metabolomics Data. Anal Chem.

[CR146] Spande TF, Daly JW, Hart DJ (1981). The structure of gephyrotoxin (GTX) 223AB. Experientia.

[CR147] Spande TF, Garraffo HM, Edwards MW (1992). Epibatidine: A novel (Chloropyridyl)azabicycloheptane with potent analgesic activity from an Ecuadoran poison frog. J Am Chem Soc.

[CR148] Spande TF, Jain P, Garraffo HM (1999). Occurrence and significance of decahydroquinolines from dendrobatid poison frogs and a myrmicine ant: Use of 1H and 13C NMR in their conformational analysis. J Nat Prod.

[CR149] Strehmel N, Hummel J, Erban A (2008). Retention index thresholds for compound matching in GC-MS metabolite profiling. J Chromatogr B Anal Technol Biomed Life Sci.

[CR150] Stuckert AMM, Saporito RA, Venegas PJ, Summers K (2014). Alkaloid defenses of co-mimics in a putative Müllerian mimetic radiation. BMC Evol Biol.

[CR151] Stynoski JL, Noble VR (2012). To beg or to freeze: Multimodal sensory integration directs behavior in a tadpole. Behav Ecol Sociobiol.

[CR152] Stynoski JL, Torres-Mendoza Y, Sasa-Marin M, Saporito RA (2014). Evidence of maternal provisioning of alkaloid-based chemical defenses in the strawberry poison frog *Oophaga pumilio*. Ecology.

[CR153] Sud M, Fahy E, Cotter D (2016). Metabolomics Workbench: An international repository for metabolomics data and metadata, metabolite standards, protocols, tutorials and training, and analysis tools. Nucleic Acids Res.

[CR154] Summers K, Clough ME (2001). The evolution of coloration and toxicity in the poison frog family (Dendrobatidae). Proc Natl Acad Sci.

[CR155] Tarvin RD, Santos JC, O’Connell LA (2016). Convergent substitutions in a sodium channel suggest multiple origins of toxin resistance in poison frogs. Mol Biol Evol.

[CR156] Tarvin RD, Powell EA, Santos JC (2017). The birth of aposematism: High phenotypic divergence and low genetic diversity in a young clade of poison frogs. Mol Phylogenet Evol.

[CR157] Tokuyama T, Daly JW (1983). Steroidal alkaloids (batrachotoxins and 4β-hydroxybatrachotoxins), “indole alkaloids” (calycanthine and chimonanthine) and a piperidinyldipyridin. Tetrahedron.

[CR158] Tokuyama T, Daly J, Witkop B (1968). The structure of batrachotoxinin A, a novel steroidal alkaloid from the Colombian arrow poison frog, *Phyllobates aurotaenia*. J Am Chem Soc.

[CR159] Tokuyama T, Uenoyama K, Brown G (1974). Allenic and acetylenic spiropiperidine alkaloids from the neotropical frog, *Dendrobates histrionicus*. Helv Chim Acta.

[CR160] Tokuyama T, Daly JW, Highet RJ (1984). Pumiliotoxins: magnetic resonance spectral assignments and structural definition of pumiliotoxins A and B and related allopumiliotoxins. Tetrahedron.

[CR161] Tokuyama T, Nishimori N, Shimada A (1987). New classes of amidine, indolizidine and quinolizidine alkaloids from a poison-frog, *Dendrobates pumilio* (Dendrobatidae). Tetrahedron.

[CR162] Twomey E, Johnson JD, Castroviejo-Fisher S, Van Bocxlaer I (2020). A ketocarotenoid-based colour polymorphism in the Sira poison frog *Ranitomeya sirensis* indicates novel gene interactions underlying aposematic signal variation. Mol Ecol.

[CR163] Twomey E, Kain M, Claeys M (2020). Mechanisms for color convergence in a mimetic radiation of poison frogs. Am Nat.

[CR164] Vaelli PM, Theis KR, Williams JE (2020). The skin microbiome facilitates adaptive tetrodotoxin production in poisonous newts. Elife.

[CR165] Van den Berg RA, Hoefsloot HCJ, Westerhuis JA (2006). Centering, scaling, and transformations: Improving the biological information content of metabolomics data. BMC Genomics.

[CR166] Vandendriessche T, Abdel-Mottaleb Y, Maertens C (2008). Modulation of voltage-gated Na+ and K+ channels by pumiliotoxin 251D: A “joint venture” alkaloid from arthropods and amphibians. Toxicon.

[CR167] Vences M, Kosuch J, Boistel R (2003). Convergent evolution of aposematic coloration in Neotropical poison frogs: a molecular phylogenetic perspective. Org Divers Evol.

[CR168] Walker JM (2013). Metabolomics tools for natural product discovery: Methods and protocols.

[CR169] Wang IJ (2011). Inversely related aposematic traits: Reduced conspicuousness evolves with increased toxicity in a polymorphic poison-dart frog. Evolution (n y).

[CR170] Wang M, Carver JJ, Phelan VV (2016). Sharing and community curation of mass spectrometry data with Global Natural Products Social Molecular Networking. Nat Biotechnol.

[CR171] Watrous J, Roach P, Alexandrov T (2012). Mass spectral molecular networking of living microbial colonies. Proc Natl Acad Sci U S A.

[CR172] Wells SC (2007). The ecology and behavior of amphibians.

[CR173] Wickham H (2016) ggplot2: Elegant Graphics for Data Analysis. In: Springer-Verlag New York. https://github.com/hadley/ggplot2-book. Accessed May 2021

[CR174] Willink B, Brenes-Mora E, Bolaños F, Pröhl H (2013). Not everything is black and white: Color and behavioral variation reveal a continuum between cryptic and aposematic strategies in a polymorphic poison frog. Evolution (n y).

[CR175] Xia J, Wishart DS (2016) Using metaboanalyst 3.0 for comprehensive metabolomics data analysis. Curr Protoc Bioinforma 55(1):10–14. 10.1002/cpbi.11 10.1002/cpbi.11 27603023

[CR176] Xian F, Hendrickson CL, Marshall AG (2012). High Resolution Mass Spectrometry. Anal Chem.

[CR177] Yasumoto T, Michishita T (1985). Fluorometric determination of tetrodotoxin by high performance liquid chromatography. Agric Biol Chem.

[CR178] Yotsu M, Yasumoto T, Hae Kim Y (1990). The structure of chiriquitoxin from the costa rican frog *Atelopus chiriquiensis*. Tetrahedron Lett.

[CR179] Yotsu-Yamashita M, Kim YH, Dudley SC (2004). The structure of zetekitoxin AB, a saxitoxin analog from the Panamanian golden frog *Atelopus zeteki*: A potent sodium-channel blocker. Proc Natl Acad Sci.

